# The short coiled-coil domain-containing protein UNC-69 cooperates with UNC-76 to regulate axonal outgrowth and normal presynaptic organization in *Caenorhabditis elegans*

**DOI:** 10.1186/jbiol39

**Published:** 2006-05-25

**Authors:** Cheng-Wen Su, Suzanne Tharin, Yishi Jin, Bruce Wightman, Mona Spector, David Meili, Nancy Tsung, Christa Rhiner, Dimitris Bourikas, Esther Stoeckli, Gian Garriga, H Robert Horvitz, Michael O Hengartner

**Affiliations:** 1 Institute for Molecular Biology, University of Zurich, Winterthurerstrasse 190, CH-8057 Zurich, Switzerland; 2 Neuroscience Center Zurich, ETH and University of Zurich, Winterthurerstrasse 190, CH-8057 Zurich, Switzerland; 3 Program in Genetics, SUNY at Stony Brook, Stony Brook, NY 11794, USA; 4 Cold Spring Harbor Laboratory, Cold Spring Harbor, NY 11724, USA; 5 Howard Hughes Medical Institute, Department of Molecular, Cellular and Developmental Biology, Sinsheimer Laboratories, University of California, Santa Cruz, CA 95064, USA; 6 Biology Department, Muhlenberg College, Allentown, PA 18104, USA; 7 Zoological Institute, University of Zurich, Winterthurerstrasse 190, CH-8057 Zurich, Switzerland; 8 Howard Hughes Medical Institute, Department of Biology, Massachusetts Institute of Technology, Cambridge, MA 02139, USA; 9 Department of Molecular and Cell Biology, University of California, Berkeley, CA 94720, USA; 10 Current address: Department of Neurosurgery, Brigham and Women's Hospital, Children's Hospital and Harvard Medical School, 300 Longwood Avenue, Boston, MA 02115, USA; 11 Current address: Abteilung für Klinische Chemie und Biochemie, Universitäts-Kinderklinik, Steinwiesstrasse 75, CH-8032 Zürich, Switzerland; 12 Current address: Clinigen, Inc., 400 W. Cummings Park #5700, Woburn, MA 01801, USA

## Abstract

**Background:**

The nematode *Caenorhabditis elegans *has been used extensively to identify the genetic requirements for proper nervous system development and function. Key to this process is the direction of vesicles to the growing axons and dendrites, which is required for growth-cone extension and synapse formation in the developing neurons. The contribution and mechanism of membrane traffic in neuronal development are not fully understood, however.

**Results:**

We show that the *C. elegans *gene *unc-69 *is required for axon outgrowth, guidance, fasciculation and normal presynaptic organization. We identify UNC-69 as an evolutionarily conserved 108-amino-acid protein with a short coiled-coil domain. UNC-69 interacts physically with UNC-76, mutations in which produce similar defects to loss of *unc-69 *function. In addition, a weak reduction-of-function allele, *unc-69(ju69)*, preferentially causes mislocalization of the synaptic vesicle marker synaptobrevin. UNC-69 and UNC-76 colocalize as puncta in neuronal processes and cooperate to regulate axon extension and synapse formation. The chicken UNC-69 homolog is highly expressed in the developing central nervous system, and its inactivation by RNA interference leads to axon guidance defects.

**Conclusion:**

We have identified a novel protein complex, composed of UNC-69 and UNC-76, which promotes axonal growth and normal presynaptic organization in *C. elegans*. As both proteins are conserved through evolution, we suggest that the mammalian homologs of UNC-69 and UNC-76 (SCOCO and FEZ, respectively) may function similarly.

## Background

At its simplest, a neuron is composed of three major structures, a central cell body and two networks of extensively branched membrane structures, the dendrite and the axon. Growing axons respond to a wide variety of extracellular attractive and repulsive signals that direct migration to a fated location. Although many guidance receptors have been identified on extending growth cones, little is known about how activation of receptors mediates coordinated neurite extension. In addition to signaling cues in the extracellular matrix, neurite elongation and growth-cone extension depend on a concerted effort of vesicular transport and regulated membrane addition. For growth cones to extend, vesicles derived from the Golgi apparatus fuse with the plasma membrane by a process of regulated exocytosis [1]. Likewise, synapse formation also requires transport of pre- and post-synaptic components supplied in membranous organelles [2,3]. These vesicles are not only transported but are also differentially sorted into dendrites or axons [4,5]. To fulfill these tasks, intrinsic cytosolic factors are required to regulate transport of the vesicles [6] and to differentially control dendritic versus axonal growth and morphogenesis.

The nematode *Caenorhabditis elegans *has been extensively used to study vesicular transport in neuronal development. For example, monomeric kinesin UNC-104/KIF1A, UNC-116/kinesin heavy chain (KHC), kinesin light chain KLC-2, and various cytoplasmic dynein complex components regulate various vesicle trafficking events [7-9]. KLC-2 might regulate the transport of various axonal and synaptic cargos by recruiting adaptor and regulatory proteins such as UNC-16, UNC-14 and UNC-51 [9,10]. In the absence of UNC-16 (a JNK-scaffolding protein), a glutamate receptor and synaptic vesicles containing the synaptobrevin homolog SNB-1 dislodge from the post- and pre-synaptic terminals [7]. UNC-16 binds directly to the tetratricopeptide repeat (TPR) domain of KLC-2, whereas the RUN-domain-containing protein UNC-14 associates with UNC-16 in the presence of KLC-2 [9]. UNC-14 interacts physically with the serine/threonine kinase UNC-51, and both proteins are required for axonal outgrowth [10,11]. Noticeably, although membranous structures with variable size accumulate within axons in *unc-51 *[12,13] and *unc-14 *[13] mutants, suggesting that both genes are involved in axonal transport, synaptic vesicles are normally clustered in presynaptic terminals in these mutants [13].

*C. elegans *UNC-76 and its homologs have been implicated in both axonal outgrowth and synaptic transport via association with the heavy chain of Kinesin-1. In worms mutant for *unc-76*, the nervous system is disorganized: the axons fail to extend and axonal bundles are defasciculated [13,14]. In *Drosophila*, Unc-76 interacts with the tail of KHC and is important for transporting synaptic cargos in the axons [15]. The mechanism of UNC-76-mediated transport remains elusive, although there is some evidence that secondary modification by protein kinase Cζ (PKCζ) or polyubiquitination of the fasciculation and elongation protein zygin/zeta 1 (FEZ1), one of the mammalian UNC-76 homologs, contributes to its neurite outgrowth activity [16,17].

In this study we report the cloning and characterization of UNC-69, a small, evolutionarily conserved coiled-coil domain-containing protein that acts as a novel binding partner of UNC-76 in *C. elegans *. Whereas a weak reduction-of-function allele of *unc-69 *results in a selective defect in mislocalization of a synaptic vesicle marker, strong *unc-69 *mutants show extensive defects in axonal outgrowth, fasciculation and guidance. Mutations in UNC-69 preferentially disrupt membrane traffic within axons. We show that UNC-69 and UNC-76 participate in a common genetic pathway necessary for axon extension and cooperate to regulate the size and position of synaptic vesicles in axons. Moreover, both proteins colocalize as puncta in neuronal processes. We propose that UNC-69 and UNC-76 form a conserved protein complex *in vivo *to regulate axonal transport of vesicles.

## Results

### *unc-69 *encodes a conserved short coiled-coil domain-containing protein

*unc-69 *was identified in a large-scale behavioral screen for uncoordinated (Unc) mutants [18]. *unc-69 *loss-of-function *(lf) *mutants move poorly, coil ventrally and are phenotypically similar to other coiler Unc mutants, many of which are defective in axonal outgrowth and guidance. Additionally, *unc-69 *mutant hermaphrodites lay more eggs in the absence of food than wild-type worms do (see Additional data file 1), suggesting a defect in the hermaphrodite-specific neurons (HSNs), which control egg-laying behavior.

**Figure 1:**
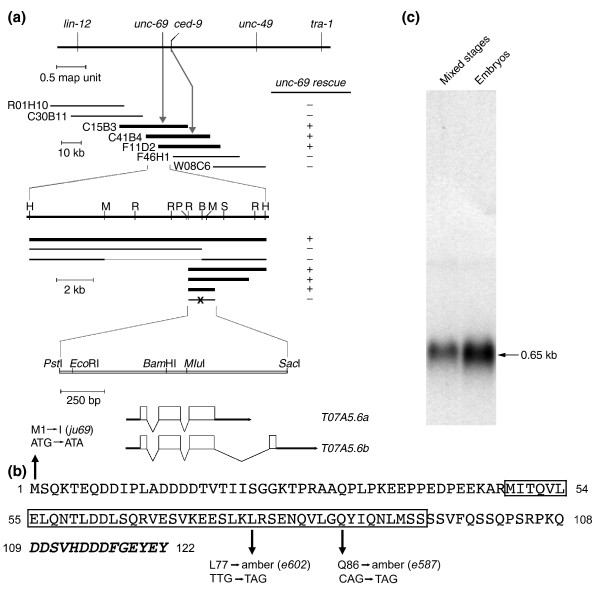
The *unc-69 *locus encodes a 108-amino-acid protein with a short coiled-coil domain. **(a) **Genetic and physical maps of chromosome III in the vicinity of the *unc-69 *locus. *unc-69 *is close to and left of *ced-9*. Cosmids and subclones able to rescue the locomotion defect of *unc-69(e587) *mutants are shown in bold. B: *Bam*HI; H: *Hin*dIII; M: *Mlu*I; P: *Pst*I; R: *Eco*RI; S: *Sac*I. Introduction of a frameshift mutation at the *Bam*HI site in the second exon (denoted with an x) abrogated rescue by the minimal *Pst*I-*Sac*I rescuing fragment. Both splice variants, *T07A5.6a *and *T07A5.6b*, are contained within this fragment. **(b) **The UNC-69 protein sequence. The boxed region is predicted to form a coiled-coil domain. Arrows indicate the positions of the three known *unc-69 *mutations. Additional amino acids encoded by *T07A5.6b *are shown in italics (see Additional data file 1). **(c) **Northern-blot analysis of *unc-69 *revealed a single major transcript of 0.65 kb (arrow).

Previous genetic data placed *unc-69 *between *lin-12 *and *tra-1 *on chromosome III, 0.12 map units to the left of *ced-9 *[19]. Using cosmid rescue, we were able to identify the predicted gene *T07A5.6a *(previously named *T07C4.10b*) as *unc-69 *(Figure 1a). The *unc-69 *gene encodes a 108-amino-acid protein and contains a short coiled-coil domain in its carboxyl terminus (Figure 1b). Although UNC-69 could possibly form a homodimer via its coiled-coil domain, we failed to detect any homophilic interactions of UNC-69 (see Additional data file 1).

**Figure 2:**
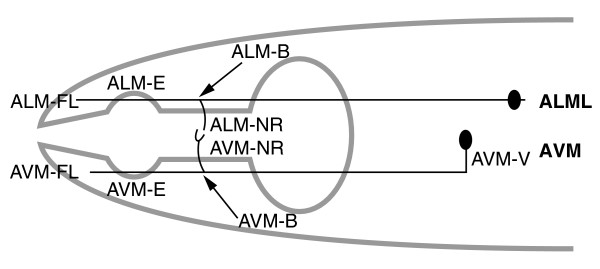
Schematic diagram of the ALM and AVM neurons in *C. elegans*. The different parts of the neurons are given designated letters; see Table 1 for details. Anterior is to the left.

The original alleles of *unc-69 *, *unc-69(e587) *and *unc-69(e602) *, are both nonsense mutations in the carboxy-terminal half of the protein (see Figure 1b). The *unc-69(e602) *mutation causes a T-to-A transversion and replaces a leucine with an amber stop codon at position 77; *unc-69(e587) *results in a C-to-T transition, changing a glutamine to an amber stop codon at position 86; both of these mutations lie within the well conserved coiled-coil domain. Both *unc-69(e602) *and *unc-69(e587) *are candidate genetic null alleles, as the axon extension and branching defects of the neurons named ALM and AVM were not enhanced significantly when either of these two alleles was placed *in trans *to the deficiency *nDf40 *(Table 1, Figure 2).

We also isolated a hypomorphic allele, *ju69*, which results in a G-to-A transition at the start codon and changes the initiator methionine to an isoleucine. Theoretically, the M-to-I substitution (M1I) should abolish translation initiation and hence synthesis of the UNC-69 protein. As the phenotype of *unc-69(ju69) *mutants is much weaker than that of the other two alleles, however, we suspect that a small amount of UNC-69 functional protein is still being produced, either by leaky translation initiation at the original site, or through initiation at the internal, in-frame ATG site at residue 49, which would leave the coiled-coil domain intact. Indeed, overexpression of a mutant fusion protein of UNC-69 with green fluorescent protein (UNC-69(M1I)::GFP) or a carboxy-terminal fragment of UNC-69 (residues 41–108) could partially suppress the locomotion defect of the *unc-69(e587) *mutants (data not shown, and see Additional data file 1).

Finally, we analyzed a small deletion, *ok339*, which completely eliminates the *unc-69 *locus. Unfortunately, this deletion also removes the essential neighboring gene *T07A5.5 *and was therefore not studied further (see Additional data file 1). Expressed sequence tag (EST) analysis suggested that the *unc-69 *locus encodes two splice variants (see Figure 1a and see Additional data file 1). Northern blot analysis of poly(A)^+ ^RNA from mixed-stage worms as well as from embryos revealed a 0.65 kb major transcript (Figure 1c), consistent with the predicted size of the *T07A5.6a *transcript.

### UNC-69 is conserved from single-celled eukaryotes to complex metazoans

**Figure 3:**
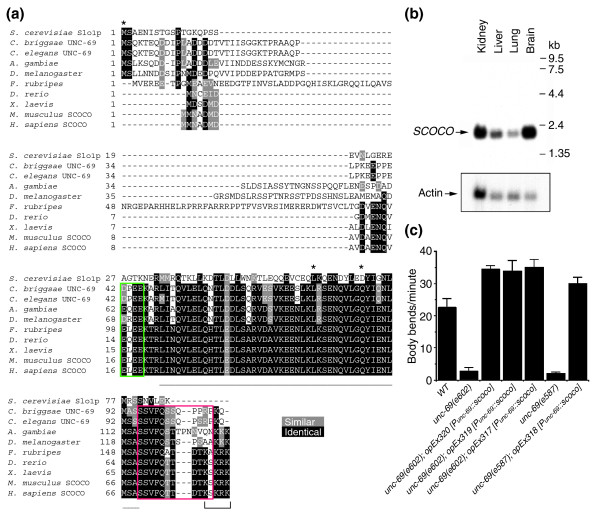
UNC-69 is homologous to mammalian SCOCO. **(a) **Sequence alignment of UNC-69/SCOCO proteins from *S. cerevisiae*, *C. elegans*, *C. briggsae*, mosquito, *Drosophila*, *Fugu*, zebrafish, *Xenopus*, mouse and human. Residues identical in all ten sequences are shaded black; similar residues are shaded gray. The underlined region is predicted in all cases to form a coiled-coil domain. The region boxed in green is acidic, and the region boxed in red is serine/threonine-rich. The bracket indicates the carboxy-terminal basic region. Asterisks mark mutations in *unc-69*. **(b) **mRNA of the human *unc-69 *homolog *SCOCO *is enriched in fetal brain and is also present in fetal kidney, liver and lung. **(c) **Expression of human *SCOCO *rescues the locomotion defect of *unc-69 *mutant. Movement of the wild type (WT), mutants, and transgenic L4-stage hermaphrodites was scored as complete sine waves per minute. For each genotype *n *= 10. Error bars represent the standard error of the mean.

We found that UNC-69 is highly conserved through evolution and encodes the *C. elegans *homolog of mammalian SCOCO (short coiled-coil protein), a protein recently found to interact with dominant-negative ARF-like 1 (ARL1) protein in a yeast two-hybrid screen [20]. The *Saccharomyces cerevisiae *UNC-69 homolog, Slo1p (SCOCO-like open reading frame protein), has been shown to interact with Arl3p, a homolog of mammalian ARFRP1, another ARF-like protein, which is involved in endoplasmic reticulum-Golgi and post-Golgi transport [21,22]. Uncharacterized UNC-69/SCOCO homologs can also be found in many other animal species (Figure 3a and Additional data file 1).

All of the UNC-69 homologs are predicted to form a coiled-coil structure near their carboxyl termini (the underlined region in Figure 3a). In an alignment of the *S. cerevisiae*, *C. elegans*, *C. briggsae*, mosquito, fly, *Fugu*, zebrafish, *Xenopus*, mouse and human protein sequences, identity over the coiled-coil regions is 32.6% (Figure 3a). The identity in the coiled-coil region jumps to 73.9% if the yeast sequence is excluded. Except for yeast, an acidic region immediately upstream of the coiled-coil domain as well as a serine/threonine-rich region and a basic region downstream appear also to be highly conserved. In contrast, the amino terminus of UNC-69 and its homologs is highly divergent, both in length and in amino-acid sequence. The function of UNC-69 proteins seems to be conserved, since expression of human SCOCO as a transgene under the *unc-69 *promoter restored locomotion to *unc-69 *mutants (Figure 3c).

We assessed the tissue distribution of human *SCOCO *transcripts by probing a human fetal tissue northern blot. This probe detected a single transcript of approximately 2.1 kb in all tissues examined (brain, lung, liver and kidney; Figure 3b). Human *SCOCO *mRNA appeared to be enriched in fetal brain, possibly hinting at a role for SCOCO in mammalian nervous system development.

### UNC-69 is expressed in the nervous system and other tissues from early embryogenesis to adulthood

**Figure 4:**
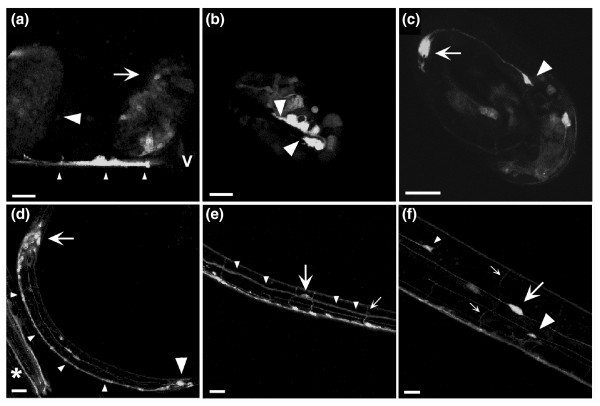
UNC-69::GFP is expressed in neurons. Confocal micrographs of mosaic animals expressing a rescuing carboxy-terminal UNC-69::GFP fusion. A 1 μm optical section is shown in **(a) **all other panels are projections of optical series. (a) Late gastrula (large arrowhead) and early comma-stage embryo (arrow) with widespread expression of UNC-69::GFP. Embryos were still inside the mother. Small arrowheads indicate the maternal VNC; v indicates the maternal vulva. **(b) **A two-fold-stage embryo with strong UNC-69::GFP expression in VNC neurons (between arrowheads). **(c) **A three-fold embryo expressing UNC-69::GFP in a growth cone (arrowhead). The arrow indicates a neuronal cell body. **(d) **An L1-stage larva expressing UNC-69::GFP in neurons and axons in the head (arrow), VNC (small arrowheads) and tail (large arrowhead). The asterisk indicates reporter expression in labial sensory neuronal processes of an adjoining adult animal. **(e) **An L3 larva expressing UNC-69::GFP in the CAN neuron (large arrow), excretory canal (small arrowheads) and in commissural axons (small arrow). **(f) **An L4 larva expressing UNC-69::GFP in the CAN (large arrow), HSN (large arrowhead) and ALM (small arrowhead) neurons. Small arrows indicate commissures. All scale bars represent 10 μm. In all cases, anterior is to the left and dorsal is up.

We generated transgenic animals expressing either amino- or carboxy-terminally *gfp*-tagged *unc-69 *fusion constructs under the control of the endogenous *unc-69 *promoter. Both translational fusion constructs rescued the Unc phenotype of *unc-69 *mutants, suggesting that the fusion proteins were correctly expressed and biologically functional. UNC-69::GFP expression was first detectable in embryos (Figure 4a,b). In immature neurons, we observed expression of UNC-69::GFP in the processes and growth cones of developing neurites (arrowhead in Figure 4c). In older larvae and adults, UNC-69::GFP was expressed in neurons of the anterior, lateral, ventral and retro-vesicular ganglia in the head, and in neurons of the preanal, dorso-rectal and lumbar ganglia in the tail. The fusion protein was also present in the ventral nerve cord (VNC), in the dorsal nerve cord (DNC), in the dorsal and ventral sublateral nerve cords, and in commissural axons (Figure 4d–f). The reporter was expressed in the neurons named CAN, HSN, ALM, PLM, AVM, PVM, BDU, and SDQR, as evidenced by its localization to the cell bodies of these neurons. Expression of *unc-69 *in these latter cells was confirmed using an *unc-69::LacZ::NLS *fusion (data not shown). Taken together, these results indicate that *unc-69 *is expressed widely, perhaps ubiquitously, in the *C. elegans *nervous system.

Expression of UNC-69::GFP was also observed in non-neuronal cells. In larvae and adults, we occasionally observed UNC-69::GFP expression in body-wall muscle (data not shown). We also observed UNC-69::GFP in the excretory canal, in the distal tip cells, in the spermatheca and, less frequently, in hypoderm and gut (Figure 4e, and data not shown). The expression in these non-neuronal cells was variable, however, and might not reflect the endogenous expression pattern of *unc-69 *.

### UNC-69 is required for axonal outgrowth and guidance

**Figure 5:**
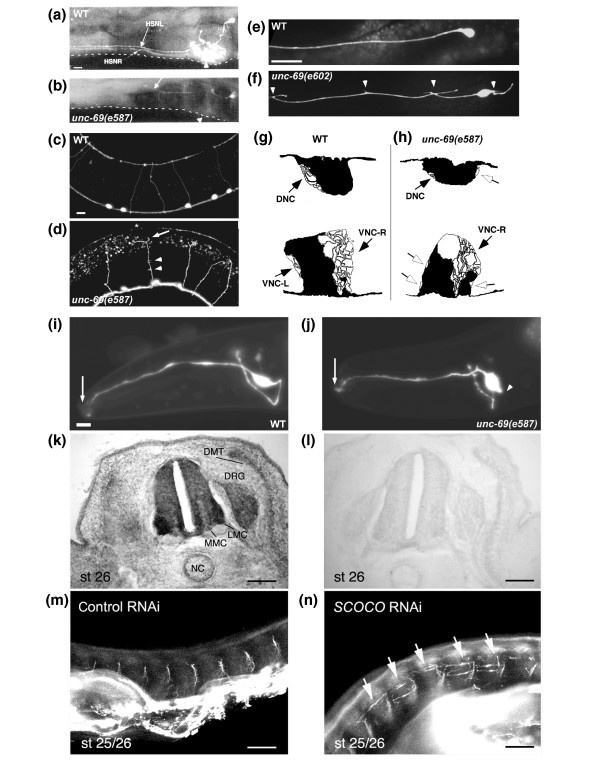
*unc-69 *is required for axonal outgrowth, guidance, branching and fasciculation in invertebrates and vertebrates. **(a,b) **Defect in the migration of the HSN neuron in *unc-69 *mutant animals. (a) In wild-type animals, the HSN axons (HSNL and HSNR) migrate ventrally until they reach the VNC, which they join and follow rostrally towards the head (arrow in (a)). (b) In *unc-69 *mutants, HSN axons occasionally fail to grow ventrally and instead project laterally along the body wall (arrow in (b)). Animals were stained with anti-serotonin antibodies to visualize the HSN neurons. Arrowheads indicate the vulva. Dotted lines mark the ventral margin of the body walls. **(c,d) **Commissures of D-type GABAergic neurons routinely reach the DNC in wild-type animals (c), but often fail in *unc-69(e587) *animals (d) and prematurely bifurcate (arrow). D-type GABAergic neurons were visualized with the *unc-47::gfp *transgene *oxIs12*. Asterisk in (d) marks a gap in the DNC. There are also often ectopic sprouts from the commissures (arrowheads in (d)) in *unc-69(e587) *mutants. **(e,f) **Images of the single ALM touch neuron in (e) wild-type and (f) *unc-69(e602) *animals. Many ectopic neurites branched out from the soma and the axonal shaft of the ALM neuron in *unc-69(e602) *mutant (arrowheads). **(g,h) **Tracings of representative electron micrographs of sections through the DNC and VNC. (g) In the wild type, the position and content of the three major fascicles are highly stereotyped (black arrows). (h) In *unc-69(e587) *mutants, defasciculated axons can often be found migrating separately along the body wall (open arrows). **(i,j) **Morphology of the bipolar AWC sensory neuron in (i) wild-type and (j) *unc-69(e587) *animals. Dendrites of AWC neurons in both animals reach the nose (arrows). Axonal shape is normal in wild-type worms, but abnormal in *unc-69(e587) *mutants, with ectopic bulges occasionally extending from the soma (arrowhead in (j)). **(k,l) **Expression pattern of *SCOCO *in stage 26 chick embryos. Sections were incubated with (k) antisense and (l) sense RNA probes for chick *SCOCO*. *SCOCO *was highly expressed in neural tissue and was most prominent in DRGs and in motoneurons of both the lateral motor column (LMC) and the medial motor column (MMC). Expression in the notochord (NC) and dermamyotome (DMT) was less pronounced. **(m,n) ***In ovo *RNAi of chick *SCOCO*. Embryos injected and electroporated with double-stranded RNA corresponding to (m) a *yfp*-containing plasmid or (n) chick *SCOCO *were immunostained with anti-neurofilament antibodies. (m) In control embryos, the epaxial nerves extending dorsally toward their target, the epaxial muscle, were highly fasciculated. (n) RNAi of *SCOCO *led to defasciculation of epaxial nerve bundles and extensive branching between muscle segments (arrows). In all panels dorsal is up. Scale bars represent: (a-j) 10 μm, (k,l) 100 μm and (m,n) 500 μm.

The ventral coiler phenotype of *unc-69 *mutants suggests a defect in nervous system development. Indeed, previous studies had reported axonal guidance defects of the D-type GABAergic motor neurons, mechanosensory neurons and the HSN neurons in *unc-69 *mutants [23,24]. We confirmed these observations and extended them to other cell types (see Tables 1, 2 and Figures 2, 5a–f). Incorrect targeting of the DD and VD motor axons is likely to contribute to the Unc phenotype of *unc-69 *mutants. In addition to outgrowth and guidance defects, we also observed ectopic branching of the DD/VD neurons and mechanosensory neurons in *unc-69 *mutants (Figure 5d,f). In a few cases the axons had unusual large swellings and occasionally meandered along the lateral body wall.

FMRF-amide (Phe-Met-Arg-Phe-NH_2_) is a neuropeptide that serves as a neuromodulator, and is co-released together with other neurotransmitters. In examining other neuronal classes in *unc-69(e587) *mutants, we observed premature termination of axons of the FMRF-amide-positive neurons ALA, RID and AVKR, but not RMG (data not shown, and see Table 2). FMRF-amide-positive neurons are so-called neuropeptidergic neurons and could be sensory, motor or interneurons. We observed that 67% (20/30) of ALA axons terminated prematurely, and ALA axons sometimes branched before termination. AVKR had frequent axonal outgrowth and guidance defects: 85% (17/20) of AVKR axons terminated prematurely or crossed from the left VNC (VNCL) to the right VNC (VNCR). Taken together, these observations support a role for *unc-69 *in ventral and dorsal axonal guidance as well as in axonal elongation within the fascicles.

### UNC-69 is required for fasciculation

As *unc-69 *mutants have midline crossover defects (see Table 2), it is likely that axons running in the same fascicle lose cell-cell adhesion and fail to stay together. We constructed a series of electron micrograph (EM) cross-sections through the major nerve cords (DNC, VNCL and VNCR) that run antero-posteriorly in adult hermaphrodites. In wild-type animals, the composition of axons in any of these nerve cords is highly stereotyped, with four axons fasciculated to run in VNCL and the other ventral axons running within VNCR (Figure 5g) [25]. In *unc-69(e587) *and *unc-69(e602) *mutants, many fascicles split into two or more groups and in some cases defasciculated axons could be seen running alone along the hypodermal ridge. Moreover, some axons of both the DNC and VNCL appeared to be mislocalized and can be seen on the wrong side of the hypodermal ridge (Figure 5h and data not shown). Anti-tubulin and anti-GABA staining confirmed the observed fasciculation defects in *unc-69(e587) *mutants (data not shown).

### UNC-69 acts cell autonomously to control neurite outgrowth

To determine whether *unc-69 *expression is required in the growing neurites or in the surrounding tissues, we created *unc-69 *transgenic lines expressing *unc-69(+) *specifically in the six touch neurons under the control of a *mec-7 *promoter. We compared outgrowth and guidance defects of the ALM and AVM neurons in three such lines with those of *unc-69(lf) *mutants (see Table 1, Figure 2). In all three transgenic lines, the percentage of ALM neurites that failed to extend to full length or send a branch into the nerve ring dropped significantly. Similar observations were made for AVM outgrowth and branching. Note that none of the transgenic lines completely rescued the ALM outgrowth and branching defects. This could be due to loss or silencing of the transgene carried on the extrachromosomal array or could reflect a requirement for *unc-69 *in other neuronal and/or non-neuronal cells. Nevertheless, we conclude that UNC-69 promotes outgrowth and guidance largely, if not completely, in a cell-autonomous manner.

### UNC-69 is required for normal presynaptic organization

**Figure 6:**
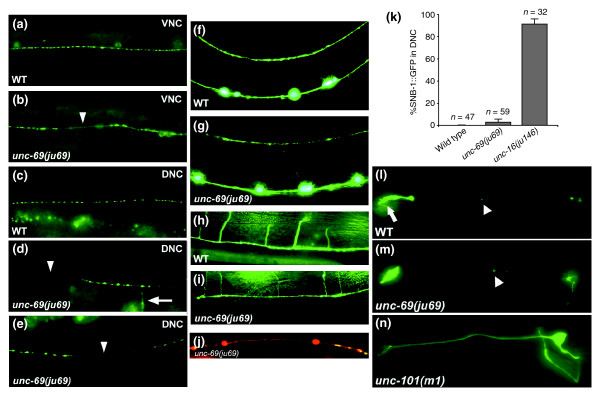
*unc-69 *affects axonal but not dendritic trafficking. **(a,c) **SNB-1::GFP is seen as evenly spaced puncta along the (a) VNC and (c) DNC in wild-type animals. **(b,d,e) **In *unc-69(ju69) *mutants, SNB-1::GFP puncta are on average bigger and often are absent from the VNC (arrowhead in (b)) and the DNC (arrowheads in (d,e)). In addition, SNB-1::GFP sometimes diffuses into the commissure (arrow in (d)). (a,b,e) Lateral views; (c,d) dorsal views of adult hermaphrodites. **(f-i) **As in (f,h) wild-type animals , neuronal morphology is grossly normal in (g,i) *unc-69(ju69) *mutants, and commissures still routinely reach the DNC. D-type GABAergic neuron morphology is visualized with the *P*_*unc*-25_*::gfp *transgene *juIs76*. (f,g) Lateral views; (h,i) dorsal views. (j) Distribution of SNB-1::GFP puncta in a stretch of axon labeled with *P*_*unc*-25_*::DsRed monomer *in the DNC in a *unc-69(ju69) *mutant hermaphrodite. SNB-1::GFP puncta are unevenly distributed, even though the DNC anatomy is grossly normal. **(k) **SNB-1::GFP is not significantly mislocalized into DD dendrites in *unc-69(ju69) *mutants. Animals carrying an *snb-1::gfp *transgene were scored at the L1 larval stage. Whereas 90% of *unc-16(ju146) *L1 larvae (*n *= 32) show dorsal GFP, 0% of wild-type L1s (*n *= 47) and 3% *unc-69(ju69) *L1s (*n *= 59) show dorsal GFP. Error bars represent the standard error of the mean. **(l-n) **The diacetyl odorant receptor ODR-10::GFP is targeted efficiently into AWB cilia both in (l) wild-type worms and in (m) *unc-69(ju69) *mutants. (n) In contrast, ODR-10::GFP becomes diffused in the dendritic targeting mutant *unc-101*. The arrow indicates the cilia; arrowheads indicate packets of ODR-10::GFP that shuttle in the dendrites. Anterior is to the left and dorsal is up.

The *C. elegans *synaptobrevin/vesicle-associated membrane protein (VAMP) homolog SNB-1 is a vesicular soluble *N*-ethylmaleimide-sensitive factor attachment protein receptor (v-SNARE) on synaptic vesicles (SVs). Tagged SNB-1 can be used to follow SVs as they are transported to presynaptic regions [26]. We isolated an allele of *unc-69*, *ju69*, in a visual genetic screen for mislocalization of a SNB-1::GFP reporter in D-type GABAergic motor neurons. In wild-type worms, SNB-1::GFP expressed in the D neurons can be localized to discrete puncta along the VNC and DNC, at sites of neuromuscular junctions (Figure 6a,c). In *unc-69(ju69) *mutant nerve cords, SNB-1::GFP puncta were irregular in size and position, on average larger than in wild type, and often completely missing for extended stretches (Figure 6b,d,e). In addition, we occasionally observed puncta that abnormally diffused from the nerve cords into the commissures (Figure 6d). Despite the abnormal shape and distribution of presynaptic regions, the overall morphology of DD and VD neurons was grossly normal (Figure 6f–i) and only occasionally (<10%; *n *= 50) did one commissure fail to exit the VNC. We made similar observations in touch neurons using worms carrying the *P*_*mec-4*_::*gfp*transgene *zdIs5 *(data not shown), a strain chosen for reconfirming findings made on D-type GABAergic motor neurons.

Much more dramatic SNB-1::GFP distribution defects were observed in the strong mutant *unc-69(e587) *(data not shown). Because of the extensive pathfinding defects observed in strong *unc-69 *mutants, however, which might complicate interpretation of the SNB-1::GFP distribution defect, we restricted our subsequent analysis to the *unc-69(ju69) *background, in which axonal guidance is largely normal. Indeed, although *unc-69(ju69) *mutant worms are Unc, they move much better than strong *unc-69 *mutants. Thus, the locomotion defect observed in *unc-69(ju69) *mutants is probably a consequence of a defect in transport or localization of axonal cargos rather than in axon guidance.

### UNC-69 is not required for dendritic growth or for targeting proteins into dendrites

To determine whether the outgrowth defects we observed in *unc-69 *mutants are specific to axons, we examined the morphology of the AWC class of sensory neurons using the *kyIs140 *[*P*_*str*-2_*::gfp*] transcriptional reporter, which is normally stochastically activated in either the right or left AWC neuron [27]. The bilaterally symmetric AWC neurons have a distinct bipolar structure, with a dendrite extending to the tip of the nose and an axon extending into the nerve ring (Figure 5i). In *unc-69(e587) *mutants, the axon of the AWC neuron often stopped prematurely (Figure 5j), and *str-2::gfp *expression was often silenced (see below). In contrast, the dendrite of the AWC neuron had no outgrowth defect, as 100% (136/136) of the AWC dendrites extended to their full length. In *unc-69(e587) *mutants, 73% (99/136) of AWC neurons had ectopic bulges or branches protruding from either the cell body or the axon (similar to what we observed in the mechanosensory neurons, Figure 5f,j). Ectopic branches only rarely extended from dendrites, however (data not shown). Dendritic morphology was also normal in the ASI neurons (visualized by the *str-3::gfp *transgene), the AWB, AWC, ASG, ASI, ASK, and ASJ neurons (visualized by the *tax-Δ::gfp *transgene) [28,29], and the sensory neurons ASJ, ASH, ASI, ASK, ADL, and ADF (visualized by staining with the lipophilic dye DiI; data not shown). Finally, an odorant receptor was still properly localized to the cilia (see below). From these observations, we conclude that UNC-69 is probably not required for either cilia formation or dendritic elongation within the amphid sensilla, a sensory organ within the head of a worm.

In vesicle-trafficking mutants such as *unc-16* and *unc-116*, markers for synaptic vesicles are also mis-sorted into dendrites [7]. We wondered whether *unc-69 *mutants also show such a general sorting defect, or whether *unc-69 *might be required more specifically for efficient trafficking within the axons. At the L1 larval stage, the thirteen VD neurons are not yet born, and the six DD neurons are the only D-type GABAergic motor neurons present in the VNC. At this stage, the DD neurons receive their synaptic inputs from the DNC and output onto the ventral body-wall muscles. In wild-type L1s, therefore, the SNB-1::GFP puncta can be seen only along the VNC. In *unc-69(ju69) *mutants, the synaptic GFP was not significantly mislocalized to the DNC (3.4%; *n *= 59; Figure 6k). In contrast, SNB-1::GFP puncta were frequently seen in the DNC in *unc-16(ju146) *mutant L1s (90.6%; *n *= 32; Figure 6k). We also made similar observations in worms carrying a *snb-1::gfp *transgene expressed in a pair of ASI sensory neurons, in which SNB-1::GFP was not significantly mislocalized to the ASI dendrites in *unc-69(ju69) *mutants (C-W.S., Y.J. and M.O.H., unpublished data).

We next asked whether UNC-69 has any role in transporting proteins within the dendrites. We used an *odr-10::gfp *transgene that is expressed in the AWB neurons to answer this question [30]. ODR-10 is an odorant receptor for diacetyl, and is actively transported in vesicles from the cell bodies to the cilia at the end of the dendrites, where the GFP fusion is deposited (Figure 6l). In dendritic targeting mutants, such as *unc-101 *(which encodes the homolog of AP1 μ1 clathrin adaptor protein), ODR-10::GFP is not targeted to the AWB cilia [30] (Figure 6n); in contrast, in both *unc-69(ju69) *and *unc-69(e587) *mutants, ODR-10::GFP was still properly targeted (Figure 6m; data not shown). Taken together, our results suggest that dendritic development and transport of proteins into dendrites is not impaired in *unc-69 *mutants. Thus, UNC-69 is possibly specifically required for axonal transport and outgrowth.

### UNC-69 interacts physically with UNC-76

**Figure 7:**
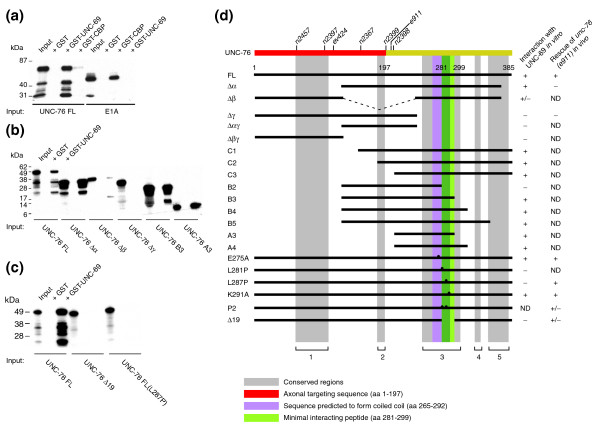
UNC-69 physically interacts with UNC-76, as shown by *in vitro *GST pull-down assays. **(a)** Full-length UNC-76 (UNC-76 FL) specifically binds to full-length GST-UNC-69 but not GST-CBP. The E1A-CBP interaction was used as a positive control. **(b)** Serial deletions of UNC-76: a portion of the carboxy-terminal region (deleted in UNC-76 Δγ but contained within UNC-76 B3 and A3) is necessary for interaction with GST-UNC-69. **(c)** Point mutation L287P or a small 19-amino-acid deletion (UNC-76 Δ19), which deletes amino acids 281–299, totally abolishes the ability of UNC-76 to bind GST-UNC-69. **(d)** Summary of the deletion analysis, as well as the results of rescuing experiments. Gray shading indicates conserved regions. Note that UNC-76 Δ19 not only loses its binding ability but also its rescuing activity for the *unc-76(e911) *mutants. The 19-amino-acid region (green) lies within a conserved region and overlaps with a region we predicted to form a coiled-coil domain (purple). A previously described axonal targeting sequence [14] is in red. The positions of different *unc-76 *alleles are indicated.

To identify potential UNC-69 interactors, we screened three *C. elegans *yeast two-hybrid libraries using full-length UNC-69 as bait. From these screens, we isolated at least 34 independent clones of UNC-76, a 385-amino-acid protein that was previously shown to be involved in axonal outgrowth and fasciculation in *C. elegans *[12-14]. The *Drosophila *homolog of UNC-76 was identified as a KHC-binding protein and shown to be a regulator of axonal transport [15]. A mammalian homolog of UNC-76, FEZ1, is a substrate for PKCζ [16]. Worm, fly and mammalian UNC-76 proteins are not only conserved in amino-acid sequence but also have several conserved regions (Figure 7d) predicted to be capable of forming coiled-coil domains [14,15]. UNC-76 localizes to axons, and worms harboring mutations in *unc-76 *have a severe Unc phenotype and coil ventrally, phenotypes very similar to those observed in *unc-69 *mutants [14].

We used an *in vitro *glutathione *S*-transferase (GST) pull-down assay to verify the physical interaction between UNC-69 and UNC-76. As shown in Figure 7a, *in vitro *translated full-length UNC-76 (UNC-76FL) was pulled down efficiently by GST-UNC-69 but only minimally by GST-CBP, a eukaryotic transcription factor used as a negative control [31]. Conversely, *in vitro *translated adenoviral protein E1A efficiently bound to its cognate partner GST-CBP but not to GST-UNC-69. Therefore, the interaction between UNC-76 and UNC-69 is specific and most likely direct.

To narrow down the regions of interaction, we generated truncated proteins lacking various parts of UNC-76 (Figure 7b,d) and tested for their interaction with GST-UNC-69. We found that amino acids 281 to 299 of UNC-76 were necessary to interact with UNC-69 *in vitro *. Interestingly, this 19-amino-acid region overlaps with a region predicted to form a coiled-coil structure (amino acids 265–292; purple region in Figure 7d) and lies within a region conserved from worms to humans (gray-shaded region in Figure 7d).

### UNC-76 may require interaction with UNC-69 to function *in vivo*

To corroborate the *in vitro *interactions with the *in vivo *function of UNC-76, we expressed truncated UNC-76 proteins tagged with yellow or cyan fluorescent protein (YFP or CFP) in *unc-76(e911) *mutant worms (Figure 7d) and assayed for rescue of the Unc phenotype. Both amino-terminally and carboxy-terminally tagged full-length UNC-76::YFP or CFP::UNC-76 fusion proteins were functional and rescued *unc-76(e911) *mutants (Figure 7d). The CFP::UNC-76 Δα fusion protein (which lacked the amino terminus of UNC-76) failed to rescue *unc-76(e911) *mutants, suggesting that the amino-terminal region of UNC-76 is required for its function *in vivo*. Bloom and Horvitz reported that amino acids 1–197 of UNC-76 are sufficient to direct proteins into the axons in *C. elegans *[14]. As the axonal targeting sequence of UNC-76 includes the region deleted in UNC-76 Δα, we speculated that CFP::UNC-76 Δα fusion proteins were not transported to axons. Indeed, the CFP signal was weak and seemed to congregate more around the soma (data not shown). In contrast, the CFP::UNC-76 Δγ fusion protein was both strongly expressed in soma and axons, but failed to rescue *unc-76(e911) *mutants, consistent with the hypothesis that binding to UNC-69 is critical for UNC-76 to function *in vivo*.

If coiled-coil structures are important for the UNC-76-UNC-69 interaction, any mutation that abolishes the coiled-coil structure would possibly also abolish physical interaction between the two proteins. To test this idea, we mutagenized four conserved residues in UNC-76: Glu275, Leu281, Leu287, and Lys291. Both UNC-76(E275A) and UNC-76(K291A) mutant proteins still bound UNC-69 *in vitro *(Figure 7d). Likewise, YFP fusions of these mutant proteins rescued *unc-76(e911) *mutants. In contrast, both UNC-76(L281P) and UNC-76(L287P) mutant proteins failed to bind UNC-69 *in vitro*. Surprisingly, UNC-76(L287P) was still able to rescue *unc-76(e911) in vivo *(Figure 7c,d; we did not test UNC-76(L281P) for rescue). These data suggest that a single-amino-acid substitution might not be potent enough to destroy the coiled-coil structure when UNC-76 protein is folded in its native state. Finally, we created a mutant protein carrying both L281P and L287P mutations (P2), as well as an internal deletion mutant, Δ19, which deletes amino acids 281–299 of UNC-76. Both P2 and Δ19 mutants largely failed to rescue *unc-76(e911) in vivo *(Figure 7d; occasionally, mutant hermaphrodites carrying the *unc-76 P2::yfp *or the *unc-76 Δ19::yfp *transgenes were slightly rescued as young adults). In summary, amino acids 281–299 of UNC-76 probably contain or overlap with an UNC-69-binding site, and UNC-76 may require interaction with UNC-69 to function *in vivo*.

### UNC-69 and UNC-76 act in the same pathway to control axon extension

As both UNC-69 and UNC-76 are required for axon outgrowth and fasciculation, we asked whether they function in the same genetic pathway to regulate axon extension. We first tested whether overexpression of UNC-69 in *unc-76(lf) *mutants could bypass the *unc-76 *mutant phenotype. We overexpressed a functional *unc-69::gfp *transgene as an extrachromosomal array in *unc-76(e911) *mutants but did not see any rescue in locomotion (three independent lines, data not shown). Likewise, overexpression of a functional *unc-76::yfp *transgene failed to rescue the locomotion defect of *unc-69(e587) *mutants (data not shown).

We also performed a double-mutant analysis to further address the question of whether *unc-69 *and *unc-76 *act in the same pathway. In *C. elegans*, expression of the odorant receptor gene *str-2 *is randomly turned on in either the left or the right AWC sensory neuron (AWCL/R), but never in both [27]. In wild-type worms, this '1 AWC^ON^' phenotype is determined by axonal contact and calcium signaling between AWCL and AWCR. In axonal guidance mutants such as *unc-76*, *sax-3 *and *vab-3*, the two AWC axons often fail to meet, and *P*_*str*-2_*::gfp *expression is consequently silenced in both AWCs, giving rise to a '2 AWC^OFF^' phenotype [27]. We used this system to quantitatively score axon extension defects in the nerve ring in different *unc-69(lf) *and *unc-76(lf) *mutants as well as in *unc-69(lf); unc-76(lf) *double mutants.

In both strong loss-of-function mutants, *unc-69(e602) *and *unc-69(e587)*, 30–34% of animals showed a 2 AWC^OFF ^phenotype. In contrast, the hypomorphic allele *unc-69(ju69) *resulted in only 1% of mutant worms (*n *= 190) having *P*_*str*-2_*::gfp *expression silenced in both AWCs (Table 3). This result was consistent with our previous observation that neuronal morphology is largely normal in *unc-69(ju69) *mutants. In agreement with previous studies [27], 47% of *unc-76(e911) *mutants (*n *= 101) had the 2 AWC^OFF ^phenotype; *e911 *was the strongest allele among all the nine alleles that we tested. For the other *unc-76 *alleles, the 2 AWC^OFF ^phenotype varied from 6% to 30%. Interestingly, the strength of the AWC expression defect (which is an indication of axon extension defects) showed an inverse colinear relationship with the position of each mutation in the open reading frame: the most 5' mutation, *unc-76(n2457)*, showed the least defect in axon extension, whereas alleles located most carboxy-terminally showed greater defects than alleles located close to the amino terminus (Table 3). Interestingly, we did not observe enhancement of axon extension defects in *unc-69; unc-76 *double mutants: in all cases, the defect in the double mutant was no stronger than in the stronger of the single mutants (Table 3). In contrast, axon extension defects were greatly enhanced in *unc-76(e911); sax-3(ky123), unc-76(e911); unc-6(n102) *and *unc-33(e204); unc-76(e911)*, and slightly enhanced in *unc-76(e911); vab-3(e648) *and *unc-119(ed3); unc-76(e911) *double mutants (Table 3). Because *unc-76 *alleles failed to show any additivity with the candidate null alleles *unc-69(e587) *and *unc-69(e602)*, we conclude that UNC-69 and UNC-76 probably act in the same pathway to control axon extension, at least in the case of the AWC sensory neurons.

### UNC-69 and UNC-76 regulate presynaptic organization cooperatively

**Figure 8:**
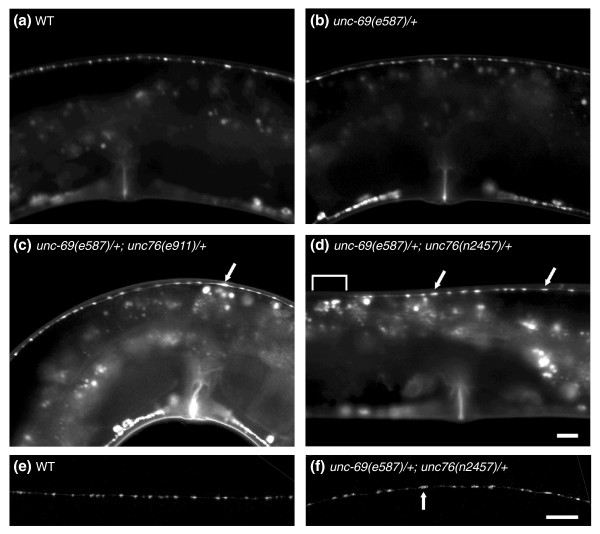
UNC-69 and UNC-76 cooperate to regulate the size and position of synaptic vesicles. **(a-d)** Lateral view of adult hermaphrodites 52–54 h after hatching, single section. **(e,f)** Lateral view of the DNC of adult hermaphrodites 52–54 h after hatching, flattened images of confocal z-stack. Anterior is to the left and dorsal is up. (a,e) SNB-1::GFP is evenly distributed along the DNC in wild-type animals. (b) Removing one copy of *unc-69 *does not affect SNB-1::GFP distribution. (c,d,f) SNB-1::GFP becomes diffused and the puncta becomes larger (arrows) in *unc-69(e587)/+; unc-76(e911)/+ *and *(unc-69(e587)/+; unc-76(n2457)/+ *double heterozygotes. Occasionally, SNB-1::GFP is missing altogether from a stretch of the DNC (bracket in (d)). The genotypes are as follows: (a,e) *juIs1 [P*_*unc*-25_*::snb-1::gfp]*, (b) *qC1/unc-69(e587); juIs1*, (c) *qC1/unc-69(e587); nT1[qIs51]/juIs1; nT1[qIs51]/unc-76(e911)*, (d,f) *qC1/unc-69(e587); nT1[qIs51]/juIs1; nT1[qIs51]/unc-76(n2457)*. Scale bars represent 10 μm.

We showed above that UNC-69 is required for localization of synaptic vesicles in axons. Does UNC-76 also have a role in this process, and if so, does UNC-76 control presynaptic organization together with UNC-69? Unfortunately, all existing *unc-76 *alleles have severe axonal outgrowth defects, making interpretations of defect in synaptic vesicle localization difficult. To bypass this problem and to reveal possible genetic interactions between *unc-69 *and *unc-76*, we looked at the localization of the synaptobrevin SNB-1::GFP puncta in *unc-69(lf)/+; unc-76(lf)/+ *double heterozygotes (Figure 8). In wild-type adult hermaphrodites, SNB-1::GFP can be seen as evenly distributed puncta along the DNC [7] (Figure 8a,e). The distribution pattern of GFP puncta in DNC was not significantly different in *unc-69(e587)/+ *heterozygotes (Figure 8b) as compared with wild-type animals. However, in both *unc-69(e587)/+; unc-76(e911)/+ *and *unc-69(e587)/+; unc-76(n2457)/+ *double heterozygous hermaphrodites, SNB-1::GFP puncta were occasionally more diffused, larger, or completely absent within a stretch of DNC (Figure 8c,d,f); the absence of SNB-1::GFP puncta may be due to either transport or axon extension defects. In addition, *unc-69(e587)/+; unc-76(e911)/+ *and *unc-69(e587)/+; unc-76(n2457)/+ *double heterozygotes occasionally had a slight Unc phenotype in locomotion, resembling weak synaptic transmission mutants. The weak locomotion defect could be a direct or indirect effect of the synaptic vesicle mislocalization defect.

In summary, the *unc-69/+; unc-76/+ *double heterozygotes show phenotypes that are similar, albeit significantly weaker, to those observed in *unc-69(ju69) *homozygotes. Haplo-insufficient genetic interactions of this type, commonly known as nonallelic (or unlinked) noncomplementation, are often observed with proteins that form heterodimers or function in a common protein complex (such as α- and β-tubulin; [32]). Several other explanations are also possible, however (discussed in [33]). Thus, our observations are compatible with, but do not definitively prove, the hypothesis that UNC-69 and UNC-76 act in a common pathway required for proper synaptic-vesicle localization.

### UNC-69 and UNC-76 colocalize in punctate structures in axons and cell bodies

**Figure 9:**
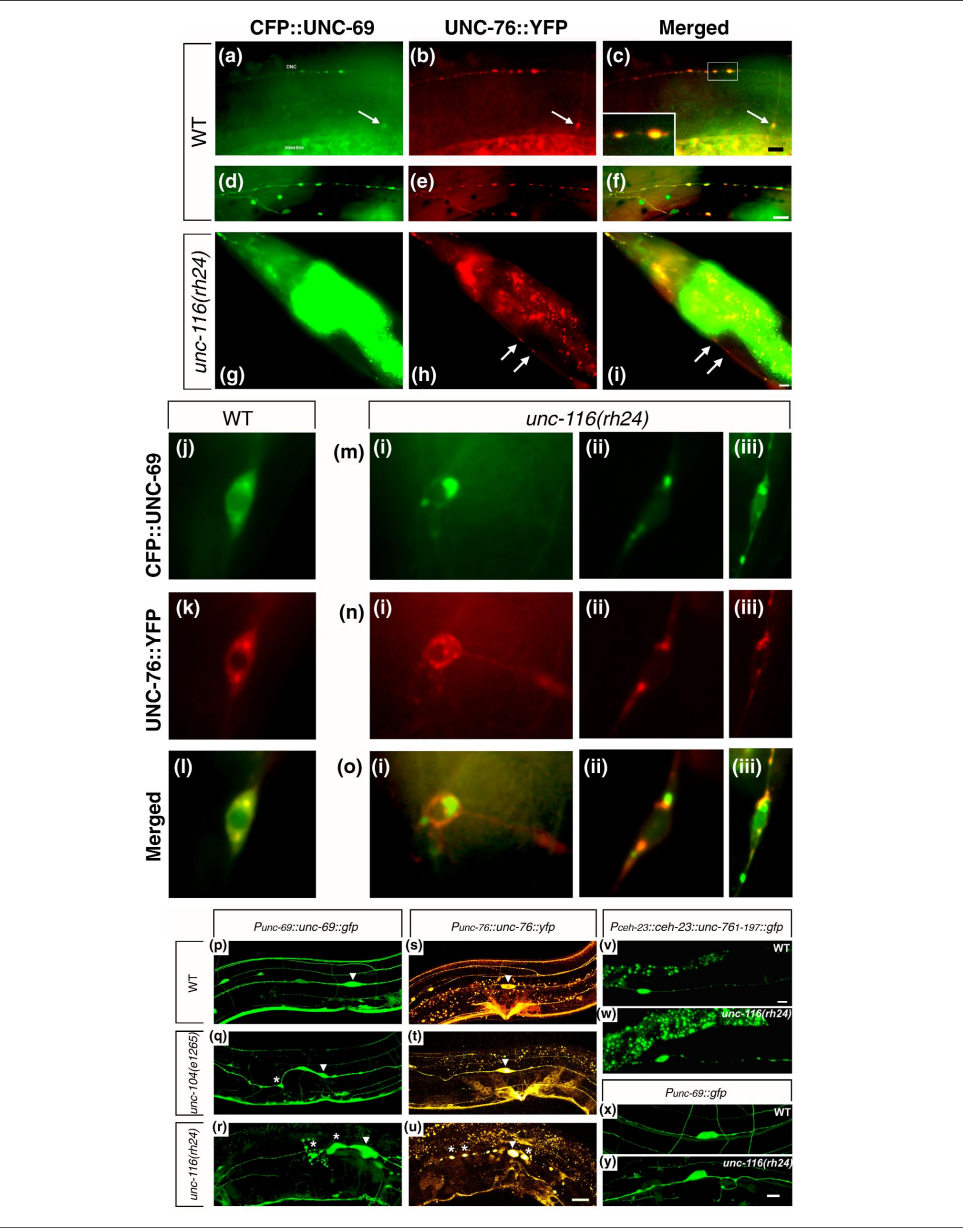
UNC-69 and UNC-76 colocalize as puncta in neuronal processes. **(a-o)** Functional *P*_*unc*-69_*::cfp::unc-69 *and *P*_*unc*-76_*::unc-76::yfp *constructs were coinjected at 5 ng/μl each into *unc-76(e911) *mutants, and worms rescued for locomotion were selected. Note that the *unc-76(e911) *mutation was removed from the background in (g-o). (d-o) are deconvoluted single-layer images. (a-c) Lateral view of an adult hermaphrodite from one line of transgenic animals with a wild-type phenotype. Both CFP::UNC-69 and UNC-76::YFP form discrete, large puncta in the DNC, as well as in the commissure (arrow). Vignette in (c) shows an enlarged image of colocalized puncta in the DNC from the rectangle. (d-f) Lateral view of an adult hermaphrodite from a second line of transgenic animals with a wild-type phenotype. Note that CFP::UNC-69 and UNC-76::YFP are both cytoplasmic and punctate, and the puncta are present in lateral and sublateral processes. (g-i) In *unc-116(rh24) *mutants, UNC-76::YFP puncta became diffuse in a stretch of axon in the VNC, and failed to colocalize with CFP::UNC-69 (arrows in (h,i)). (j-l) CFP::UNC-69 and UNC-76::YFP colocalize in perinuclear structures in the soma of a neuron in the tail ganglia. (m-o) In *unc-116(rh24) *mutants, both UNC-76::YFP and CFP::UNC-69 often appear as partially overlapping or non-overlapping aggregates in the soma of a (i) preanal  and (ii-iii) two tail ganglion neurons. **(p-u)** Expression pattern of (p-r) *opIs124 (P*_*unc*-69_*::unc-69::gfp*) and (s-u) *opIs130 (P*_*unc*-76_*::unc-76::yfp*). Both transgenes were integrated into the genome to ensure stable gene expression. All pictures show the CAN neuron soma (arrowhead) and its vicinity. (p,s) In wild-type worms, the CAN neuron extended its bipolar processes along the excretory canal, and the CAN neurites were filled with UNC-69::GFP and UNC-76::YFP. Note that puncta cannot be seen in these integrants owing to overexpression of the transgenes. (q) In *unc-104(e1265) *mutants, UNC-69::GFP accumulated near the CAN soma as well as in its neuronal processes (asterisks), giving it a notched appearance. (t) UNC-76::YFP localization appeared to be grossly normal in *unc-104(e1265) *mutants. (r,u) In *unc-116(rh24) *mutants, both UNC-69::GFP and UNC-76::YFP accumulated in CAN neurites (asterisk). UNC-69::GFP accumulation was prominent near the CAN soma and was accompanied by ectopic branches. In contrast, UNC-76::YFP aggregated and was evenly distributed along the CAN processes. The scale bar represents 20 μm. **(v,w)** The CAN neuron visualized by the integrated transgene *kyIs4 *(*P*_*ceh*-23_*::ceh-23::unc-76*_1–197_*::gfp*). (v) In wild-type worms, GFP appeared as string of dots, reminiscent of endogenous UNC-76 expression pattern. (w) In *unc-116(rh24) *mutants, GFP dots became larger and more dispersed. **(x,y)** CAN neuron visualized by an extrachromosomal array *opEx901 (P*_*unc*-69_*::gfp)*. Unlike the UNC-69::GFP fusion (r), GFP itself did not accumulate significantly around CAN soma in *unc-116(rh24) *mutants (y), although ectopic branches were frequently observed. (p-y) are confocal z-stack images. Anterior is to the left in (a-i) and (p-y); anterior is up and ventral is to the right in (j-o). All scale bars except in (p-u) represent 10 μm.

To determine the subcellular localization of UNC-69 and UNC-76, we coinjected *P*_*unc*-69_*::cfp::unc-69 *and *P*_*unc*-76_*::unc-76::yfp *constructs at low concentration (5 ng/μl) into *unc-76(e911) *mutant hermaphrodites, and selected rescued transgenic animals for examination. At low concentration, both CFP::UNC-69 and UNC-76::YFP often appeared as puncta along the DNC, in CAN neurons, as well as in other neuronal processes that run along the subdorsal and sub-ventral tracts (Figure 9a–f). Less frequently, these puncta could also be found in commissures that connect the DNC to the VNC. The punctate pattern of UNC-76 can also be observed when worms are stained with anti-UNC-76 antisera [14], consistent with this being the endogenous expression pattern of UNC-76. Both CFP::UNC-69 and UNC-76::YFP puncta were of variable size but were usually large and immobile, even in the commissures. Interestingly, CFP::UNC-69 and UNC-76::YFP proteins also colocalized in round, perinuclear dots in the soma (Figure 9j–l). These observations strengthen our belief that UNC-69 and UNC-76 coexist in a protein complex. The molecular nature of the observed UNC-69-UNC-76 puncta (multiprotein complexes or vesicles, perhaps) remains to be determined.

### UNC-116/kinesin heavy chain is required for proper subcellular distribution of both UNC-69 and UNC-76

In *Drosophila*, Unc-76 associates and copurifies with KHC, which is the major component of the conventional kinesin motor Kinesin-1 required for axonal transport towards the plus ends of microtubules [15]. A similar biochemical interaction between UNC-76 and the *C. elegans *KHC ortholog UNC-116 [34] has not been reported so far. To determine whether the UNC-69-UNC-76 complex is transported to axons by UNC-116, or by another kinesin, the KIF1A homolog UNC-104 [35], we compared the subcellular localization of both CFP::UNC-69 and UNC-76::YFP in wild-type and in different kinesin mutant backgrounds.

In *unc-116(rh24) *mutants, UNC-76::YFP puncta were occasionally diffuse and sometimes failed to be accompanied by CFP::UNC-69 puncta in a stretch of axon (Figure 9g–i). In addition, both CFP::UNC-69 and UNC-76::YFP proteins often occupied distinct but partially overlapping perinuclear territories in the soma in *unc-116(rh24) *mutants (Figure 9m–o). Whereas perinuclear CFP::UNC-69 dots increased in size in *unc-116(rh24) *mutants, perinuclear UNC-76::YFP either split into several smaller dots (as in Figure 9n(i)) or formed an irregular reticular structure (as in Figure 9n(iii)) in *unc-116(rh24) *mutants. The *unc-116(rh24) *mutants carry two missense mutations (I304M and E338K) at the end of the motor domain of KHC (amino acids 1–358) [34]. Thus, these mutations are likely to affect the processivity of KHC and cargo transport along the microtubules.

We also generated functional integrated UNC-69::GFP and UNC-76::YFP transgenes that were stably overexpressed in the nervous system and studied their subcellular localization in different kinesin mutant backgrounds. The CAN neurons are a pair of bilaterally symmetric neurons that send processes antero-posteriorly along the excretory canal (Figure 9p) [36]. In wild-type animals, UNC-69::GFP and UNC-76::YFP could be observed both in the CAN soma and throughout the processes (Figure 9p,s). In worms mutant for *unc-104(e1265)*, the *C. elegans KIF1A *homolog [35], subcellular distribution of UNC-69::GFP and UNC-76::YFP was not significantly altered (Figure 9q,t). In *unc-116(rh24) *mutants, overexpression pattern of UNC-69::GFP and UNC-76::YFP were both significantly different from wild-type animals. The CAN neuron accumulated UNC-69::GFP in the vicinity of its cell body, which was swollen and deformed. In addition, there were ectopic branches near the cell body, and UNC-69::GFP also accumulated in these processes (Figure 9r). Unlike UNC-69::GFP, UNC-76::YFP appeared as giant dots along the CAN processes in *unc-116(rh24) *mutant, as if UNC-76::YFP was removed from the cytoplasm and concentrated in certain subcellular compartments (Figure 9u). Moreover, a CEH-23::UNC-76_1–197 _::GFP fusion protein [37] also appeared as large aggregates along CAN processes in *unc-116(rh24) *mutants (Figure 9w).

In summary, our data show that the subcellular distribution of both UNC-69 and UNC-76 is altered in *unc-116(rh24) *mutants. It is striking that the nearly perfect co-localization of UNC-69 and UNC-76 is disrupted in *unc-116 *mutants. We are still at a loss to explain the molecular basis of this unexpected finding. What is clear, however, is that axonal transport of UNC-69 and UNC-76 is still occurring in *unc-116(rh24) *mutants. Thus, other kinesin motors and/or additional factors probably contribute to transport of UNC-69 and UNC-76 along the axons.

### UNC-69 does not interact with ARL-1, ARL-3, or ARFRP

**Figure 10:**
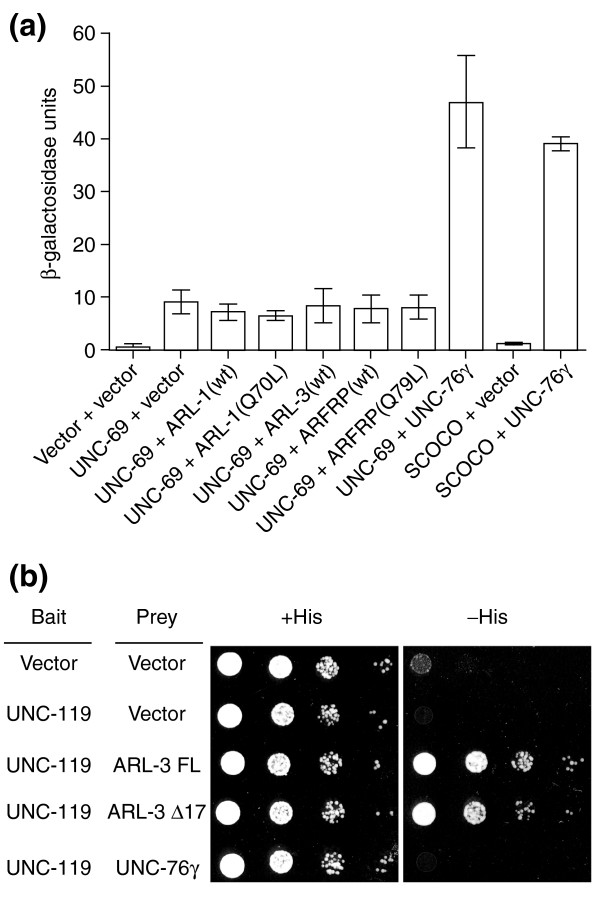
UNC-69 does not interact with ARL-1, ARL-3 or ARFRP. **(a)** Plasmids containing *LexA-unc-69 *or *LexA-human SCOCO *were cotransformed into yeast cells with vector alone or vectors containing *GAD-unc-76γ*, *GAD-arl-1*, *GAD-arl-3*, or *GAD-arfrp*. Protein-protein interactions were measured as β-galactosidase activity by using ONPG liquid assays. UNC-69 did not interaction with any of the three ARL proteins. SCOCO did not interact with any of the three ARL proteins either (data not shown). **(b)** Auxotrophic growth assays for interactions between LexA-UNC-119 and GAD-ARL-3 or GAD-UNC-76γ. Cells (3 × 10^4^) were plated onto +His or -His plates, and serial tenfold dilutions of cells were then subsequently plated. Cells were grown at 30°C for 48 h before images were taken. Note that the -His plate did not contain 3-amino triazol. The strength of interaction between UNC-119 and ARL-3Δ17 was only a fifth of that between UNC-119 and ARL-3 FL, as assayed by β-galactosidase activity (data not shown).

UNC-69 homologs in *S. cerevisiae *and mammals have been reported to interact physically with members of the family of ARF-like small GTPases. To investigate whether a similar interaction occurs in *C. elegans*, we first used yeast two-hybrid assays to study protein-protein interactions between UNC-69 and three closely related but distinct ARF-like small GTPases, ARL-1 (F54C9.10), ARL-3 (F19H8.3), and ARFRP (Y54E10BR.2) [38]. Whereas UNC-69 readily interacted with the carboxyl terminus of UNC-76 (UNC-76γ), it did not interact with any of the three ARF-like proteins (Figure 10a). As human SCOCO was isolated as an effector for GTP-bound ARL1 [20], we also tested the ability of UNC-69 to interact with GTPase-defective forms of ARL-1 and ARFRP. UNC-69 did not interact with either ARL-1(Q70L) or ARFRP(Q79L) (Figure 10a). Deletion of the amino-terminal myristoylation site [39] also had no effect: UNC-69 did not interact with the amino-terminal deletion ARL mutants, ARL-1Δ16 (with or without the GTPase-defective mutation) or or ARL-3Δ17 (data not shown). In contrast, we readily detected the previously reported interaction between ARL-3 and UNC-119 [40], a homolog of human retinal gene 4 (HRG4) [41-43] (Figure 10b). Thus, the failure to detect any interaction between UNC-69 and the three ARF-like proteins might not have been due to inappropriate protein folding or subcellular compartmentalization in yeast.

### UNC-69 and mannosidase II occupy partially overlapping subcellular regions

**Figure 11:**
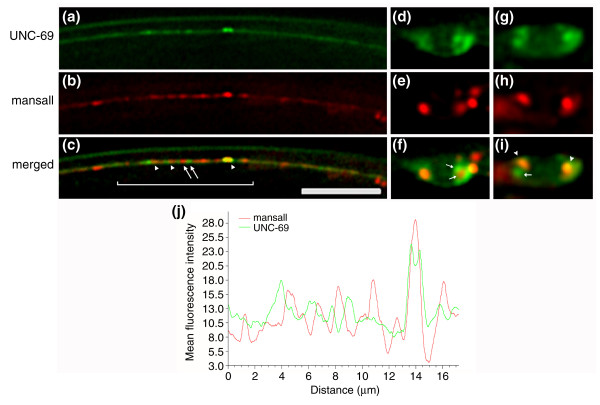
UNC-69 does not colocalize with the Golgi marker mansII. *P*_*unc*-69_*::cfp::unc-69 *and *P*_*unc*-69_*::mansII::yfp *plasmids were coinjected at 5 ng/μl each into *unc-69(e587) *mutant hermaphrodites, and worms rescued for locomotion were selected for analysis. **(a-c)** Subcellular localization of CFP::UNC-69 and mansII::YFP in a stretch of axon in the DNC in an adult hermaphrodite animal. The scale bar represents 10 μm. **(d-i)** Subcellular localization of CFP::UNC-69 and mansII::YFP in the cell bodies of a tail neuron (d-f) and a neuron in the VNC (g-i) in adult hermaphrodite animals. UNC-69 and mansII usually occupied distinct subcellular regions (arrows in (c,f,i)); only occasionally did the expression patterns of the two proteins overlap (arrowheads in (c,i)). Anterior is to the left and dorsal is up in all pictures. All pictures are deconvoluted single focal plane images. **(j)** Mean fluorescence intensity of CFP::UNC-69 and mansII::YFP along a 17 μm distance inside the bracketed region in (c).

To address directly the question of whether UNC-69 is a Golgi-associating protein, we coexpressed CFP::UNC-69 and a YFP-tagged fragment of the *C. elegans *Golgi protein mannosidase II F58H1.1 (mansII::YFP) [44]. Unlike the colocalization pattern we observed previously for UNC-69 and UNC-76, UNC-69 and mansII only occasionally colocalized (Figure 11). Moreover, we clearly observed regions in which both UNC-69 and mansII occupied non-overlapping subcellular territories, even under overexpression conditions (arrows in Figure 11c,f,i). This mutual exclusion could not simply be explained by the squeezing out of UNC-69 from the mansII-containing territories as a result of spatial constraint, as UNC-69 and mansII territories did sometimes overlap (arrowheads in Figure 11c,i).

Taken together, our results suggest that any interaction between UNC-69 and Golgi is at best transient, and that the UNC-69 puncta probably represent a structure distinct from the Golgi.

### UNC-69/SCOCO is required for axon pathfinding and fasciculation in chicken embryos

Although we failed to find any clear link between UNC-69 and Golgi-associated transport in *C. elegans*, two lines of evidence do suggest that the molecular function of UNC-69/SCOCO is conserved through evolution. First, the level of conservation between family members is extremely high in all the metazoans analyzed (see Figure 3a). Second, overexpression of human SCOCO is sufficient to rescue the uncoordinated phenotype (and hence the axon guidance defects) of *unc-69 *mutants, suggesting that human SCOCO can substitute for UNC-69 (see Figure 3c). There are, however, no reports so far on a possible role for UNC-69/SCOCO in vertebrate development. To address this issue, we studied the function of UNC-69/SCOCO in nervous system development of chicken embryos.

Expression of the chick homolog of *unc-69*/*SCOCO *was detected by *in situ *hybridization in the spinal cord of stage 22 embryos. Expression increased with time, peaking at around stage 26 (Figure 5k). In chicken embryos, SCOCO was expressed in motor neurons of both the lateral motor column (LMC) and the medial motor column (MMC). In addition to neural tissues, staining was also present in the dermamyotome (Figure 5k). Blocking the function of SCOCO with *in ovo *RNA interference (RNAi) [45] resulted in aberrant pathfinding of the epaxial nerve fibers (Figure 5n). The epaxial nerve is formed by axons of motoneurons of the MMC. These axons leave the spinal cord together with the neurons of the LMC to form the ventral root. Instead of growing into the developing limb, however, they leave the ventral root by a sharp dorsal turn. In control embryos, epaxial axons grew dorsally in a fasciculated manner and started branching only after reaching the territory of the prospective epaxial muscle (Figure 5m). In contrast, axons of epaxial motoneurons lacking UNC-69/SCOCO were strongly defasciculated and started to extend along the longitudinal axis of the embryo before reaching their dorsal destination (81% of *unc-69/SCOCO *RNAi treated embryos (*n *= 26) showed defects, versus 10% of control embryos (*n *= 20); Figure 5n and Additional data file 1). Because our *in ovo *RNAi approach selectively knocks down UNC-69/SCOCO expression in the spinal cord neurons, we conclude that the chick homolog of UNC-69/SCOCO is likely to function autonomously in epaxial nerve cells to control axon pathfinding, consistent with our observations in worms.

From the above analysis, we conclude that the function of UNC-69/SCOCO in axon guidance and nervous-system development is probably conserved through evolution. On the basis of its high degree of sequence conservation and its expression pattern, we predict that SCOCO is also required for nervous system development in mammals, including humans.

## Discussion

### UNC-69 is required for normal presynaptic organization and axonal outgrowth

In this work we show that mutations that affect the small 108-amino-acid protein UNC-69 abrogated a spectrum of processes, including synaptic-vesicle targeting, axonal outgrowth, pathfinding, and fasciculation. Although a weak reduction-of-function allele of *unc-69 *results in a selective defect in synaptic vesicle localization, strong *unc-69 *mutants have extensive axonal outgrowth, fasciculation and guidance defects. Both of the strong *unc-69(lf) *alleles, *unc-69(e602) *and *unc-69(e587)*, truncate the coiled-coil domain of UNC-69. In contrast, the hypomorphic allele *unc-69(ju69) *results in a missense mutation of the start codon and presumably interferes with translation initiation. The lack of extensive axonal outgrowth defects in the *unc-69(ju69) *mutants suggests that UNC-69 protein translation might not be totally abolished and enough UNC-69 protein is still being produced to meet the requirement for growth-cone extension. In contrast, the process of proper localization of synaptic vesicles appears to be more sensitive to reduction in levels of UNC-69 protein. It is possible that *unc-69 *mediates different cellular processes in parallel: axonal outgrowth, fasciculation and guidance on one hand, synaptic vesicle localization on the other.

UNC-69 is clearly different from most other molecules that have been implicated in axon outgrowth and guidance, which are either guidance cues, membrane receptors, or cell-adhesion proteins [46,47]. It is tempting to place UNC-69 downstream of these molecules, acting possibly as an integrator or transducer of extracellular guiding signals. Alternatively, and perhaps more likely, the axonal-outgrowth and fasciculation defects observed in strong *unc-69(lf) *mutants could be secondary to a more general transport defect, as loss of UNC-69 function might interfere not only with the transport of synaptic cargos but also with the transport of axonogenic vesicles to growth cones.

### UNC-69 and UNC-76 interact *in vivo*

We identified UNC-76 as an UNC-69-interacting protein. UNC-76 is required for axonal outgrowth and fasciculation in worms and its homolog in *Drosophila *is an axonal transport protein [14,15]. We identified a 19-amino-acid segment (amino acids 281–299) in UNC-76 that is necessary for its interaction with UNC-69, and possibly for its *in vivo *function. Our genetic experiments suggest that both UNC-69 and UNC-76 act in the same pathway to regulate axon extension. In addition, we found that UNC-76 and UNC-69 cooperate to regulate the size and position of SNB-1::GFP puncta, a marker of presynaptic regions. Finally, we showed that UNC-69 and UNC-76 colocalize in neurons as puncta and that their normal subcellular distribution requires UNC-116/KHC. The physical interaction, subcellular colocalization, and similar mutant phenotypes all suggest that the UNC-69-UNC-76 protein complex acts as a functional unit that promotes transport of vesicles along axons.

### A possible role for UNC-69 and UNC-76 in axonal transport?

Proper SNB-1::GFP localization in *C. elegans *requires many proteins, including UNC-116/KHC, KLC-2, UNC-14, UNC-51, as well as UNC-16, the worm homolog of *Drosophila *Sunday Driver, which serves as a scaffold protein receiving regulatory signals from the JNK pathway [7,9,48]. Both UNC-16 and UNC-14 are recruited to Kinesin-1 through their interaction with KLC-2 [9]. In UNC-116, KLC-2, UNC-14, and UNC-16 mutants, SNB-1::GFP is mislocalized from axons to dendrites. In contrast, SNB-1::GFP puncta were largely excluded from dendrites in *unc-69(ju69) *mutants, suggesting that UNC-69 is intrinsically different from these proteins. UNC-69 may function in a distinct step of axonal transport, and is possibly not involved in polarized sorting. Moreover, UNC-69 is unlikely to control general protein trafficking in neurons, as it was not required for dendritic transport of the transmembrane receptor ODR-10.

Kinesin-1 transports various cargos, including Golgi, endoplasmic reticulum, mitochondria and synaptic membrane proteins, but not synaptic vesicles, in the nervous system [34,49,50]. In contrast, UNC-104/KIF1A preferentially transports synaptic vesicle precursors [35,51]. Thus, the synaptic-vesicle marker mislocalization defects in various Kinesin-1 mutants [7,9,52,53] are probably secondary to a general defect of Kinesin-1-dependent cargo transport or other intracellular trafficking events within the axons. As subcellular localization of UNC-69 and UNC-76 is altered in the *unc-116(rh24) *mutants, but not in the *unc-104(e1268) *or *unc-104(rh43) *mutants (our unpublished observations), our data imply that UNC-69 and UNC-76 are possibly required for events other than transporting synaptic vesicles.

Indeed, the observation that *Drosophila *UNC-76 is a KHC-associating protein further suggests that the UNC-69-UNC-76 protein complex might constitute an alternative pathway for Kinesin-1-mediated transport. Association of the UNC-69-UNC-76 protein complex with the tail of KHC could either provide additional levels of regulation, or allow intracellular trafficking of a different repertoire of cargos. So far, however, we could not verify a direct physical interaction between *C. elegans *UNC-76 and the tail region (amino acids 696–815) of UNC-116 *in vitro *(C-W.S. and M.O.H., unpublished results). Further analysis will be required to definitively address this issue.

### Implication of UNC-69 in mediating post-Golgi transport

UNC-69 is not predicted to have enzymatic activity and possibly functions only by interacting with other coiled-coil domain-containing proteins. The budding yeast and mammalian homologs of UNC-69, Slo1p and SCOCO, have been shown to interact, respectively, with Arl3p and ARL1 - two related, Golgi-associated, GTP-binding ADP-ribosylation factor (ARF)-like proteins. Mammalian ARL1 is involved in post-Golgi transport [20]. Yeast Arl3p could target Arl1p to the Golgi, where it then tethers vesicles derived from endosomes to the Golgi [21,54]. The fact that expression of human SCOCO rescues *unc-69 *mutants suggests that a similar interaction occurs in *C. elegans*. We failed to detect any physical interaction between UNC-69 and either ARL-1 or ARFRP, however. Moreover, UNC-69 often appears to occupy subcellular regions distinct from those of the Golgi marker mansII. Thus, the exact step at which UNC-69 acts in vesicular transport (if at all) is still obscure.

### Model for UNC-69-UNC-76 protein complex in vesicular trafficking

We propose that UNC-69 and UNC-76 participate in a protein complex that is localized to certain subcellular compartments in the cytoplasm to control vesicle transport between the Golgi and the plasma membrane. The whole UNC-69-UNC-76 protein complex might be recruited and targeted by Kinesin-1 to its final destination in axons. As multiple ectopic branches were consistently observed in both *unc-69* and *unc-76 *mutants, the UNC-69-UNC-76 protein complex might also help restrict membrane addition to growth cones during neuronal development, thereby preventing unwanted membrane extension elsewhere along the axons. Similar biochemical properties of the UNC-69-UNC-76 protein complex might be used to regulate synaptic vesicle clustering or maintenance in the presynaptic regions in the adult nervous system. UNC-69-UNC-76 puncta could reflect certain post-Golgi compartments that shuttle between their budding sites on the *trans*-Golgi and their docking sites near the plasma membrane. As such, an UNC-69-UNC-76 protein complex might function at an intermediate step before vesicle maturation. Indeed, UNC-69-UNC-76 puncta were present along the commissures in addition to axons. As commissures do not form synapses [36], these puncta could define a sorting compartment from which functional vesicles are formed.

### Functional conservation of UNC-69 and SCOCO through evolution

Several lines of evidence suggest that UNC-69/SCOCO has an evolutionarily conserved role in nervous-system development in different animal species. First, human SCOCO rescues the Unc defect of *C. elegans unc-69 *mutants (see Figure 3c). Second, human SCOCO interacts with worm UNC-76γ in our yeast two-hybrid assays (Figure 10a), suggesting that the rescuing activity of human SCOCO is due at least in part to its ability to associate with UNC-76 in *C. elegans*. Third, human SCOCO and its chicken homolog are highly expressed in developing CNS neurons (see Figures 3b and 5k). Fourth, RNAi-mediated knockdown of chicken UNC-69/SCOCO results in guidance and fasciculation defects of the epaxial nerves (Figure 5m,n). It seems plausible from these observations that SCOCO also has an important role in promoting proper development (and possibly function) of the nervous system in mammals.

## Conclusion

Our studies reveal an important role for the UNC-69-UNC-76 protein complex in axonal outgrowth, fasciculation and synapse formation. Our results suggest that UNC-69 and UNC-76 act as a functional unit to regulate one or multiple steps of vesicle dynamics in the *C. elegans *nervous system. On the basis of our transgenic rescue and RNAi experiments, we suggest that vertebrates also use the UNC-69-UNC-76 complex in a similar fashion to control synapse formation and axonal outgrowth. We expect further studies to shed light on this hitherto less noticed branch of axonal guidance.

## Materials and methods

### *C. elegans *strains and genetics

*C. elegans *strains were maintained as described [18]. All strains were grown at 20°C, except *dpy-20(e1282ts) *and *lin-15(n765ts) *mutants, which were grown at 15°C before injection to improve viability and at 25°C following injection to enhance selection of transgenic F1 animals. Wild-type worms were of the Bristol N2 strain.

### Cloning of *unc-69*

All genetic mapping data were deposited into WormBase [55]. The *unc-69 *gene is tightly linked to RFLP *nP55*, which is recognized by cosmid C15B3. The three overlapping cosmids C15B3, C41B4 and F11D2, but not the flanking cosmids C30B11 and F46H1, rescued the Unc phenotype of *unc-69(e587) *mutant. Subsequent subclonings identified a 1.2-kb *Eco*RI-*Sac*I rescuing genomic fragment, which contained a single gene composed of three exons. A frameshift mutation was introduced into the *unc-69 *open reading frame of the rescuing *Eco*RI-*Sac*I fragment by cutting and filling the unique *Mlu*I restriction site, followed by re-ligation of the blunt ends. The frameshifted construct failed to rescue *unc-69 *mutant worms. To identify the molecular lesion(s) present in *unc-69 *mutants, the *unc-69 *locus from wild-type and *unc-69 *mutants was amplified using primers flanking the gene (5'-GCTCCGCAGTACGTCTTCTAAGCCC-3' and 5'-GCGAGAATGGAACAATCAATGGACG-3') and sequenced. In addition to the stop codon, *e602 *also contains a silent (third base) G-to-A transition in Lys107.

### Egg-laying assay

Assays of egg-laying behavior were performed either in M9 buffer [56] or on plates. For M9 assays, gravid hermaphrodites were individually transferred to microtiter wells containing either M9 or a 5 mg/ml solution of serotonin (5-HT, Sigma, St. Louis, USA) in M9 and the number of eggs laid after 60 min was determined. For plate assays, five gravid hermaphrodites were transferred onto fresh plates with or without food, and the total number of eggs laid after 90 min was determined.

### Immunocytochemistry and fluorescence microscopy

Indirect immunofluorescence staining for serotonin and GABA were performed as previously described [57-59]. Anti-serotonin and anti-GABA antisera were generously provided by H. Steinbusch (Free University, Amsterdam, The Netherlands) and used at 1%. Neuronal morphology was observed on a Zeiss Axioplan microscope equipped for epifluorescence, using the Zeiss filter set 488005 (excitation: 395–440 nm band-pass filter; emission: 470 nm long-pass filter). For colocalization studies, animals were anesthetized with 10 mM levamisole and mounted on 4% agarose pads in M9. A Leica DMRA2 microscope equipped with a Hamamatsu ORCA-ER CCD camera, a Leica Fluotar 40X oil objective, and appropriate filter sets was used to visualize YFP and CFP. Images were taken and deconvoluted using the Openlab software (Improvision, Coventry, UK), and analyzed using ImageJ. For confocal microscopy, a Zeiss 510 or a Leica DMRE confocal laser-scanning microscope equipped with TCS SP2 AOBS and PL APO objectives was used.

### Electron microscopy

Adult hermaphrodites were fixed in 0.8% glutaraldehyde, 0.7% OsO_4 _and 0.1 M cacodylate buffer for 1 h on ice. Subsequently, samples were cut and postfixed in 2% OsO_4 _and 0.1 M cacodylate buffer, mounted into an agar block, dehydrated in series of alcohols, and embedded in a mixture of epon-araldite. Thin sections (50 nm) were cut on an Ultracut E and pictures were taken with a JEOL 1200X at 80 kV.

### Dye filling

Adult worms were soaked in 10 μg/ml DiI (Molecular Probes, Eugene, USA) in M9, and incubated in the dark for 2 h at 20°C, followed by several washes in M9 buffer. Staining was analyzed using appropriate filters.

### cDNA screening and northern blot

A rescuing 2.8 kb *Eco*RI genomic fragment was used to probe a *C. elegans *lambdaZAP cDNA library (gift of R. Barstead). From approximately 300,000 plaques we isolated four cDNAs. The sequences present at the ends of the inserts were determined for all four clones, and the DNA sequence of the longest intact clone was determined (one strand only). A random-primed, ^32^P-labeled *unc-69 *cDNA was used to probe a northern blot of wild-type poly(A)^+ ^RNA. The blot was visualized and band intensities quantified using a Fuji Film phosphor imager. Poly(A)^+^RNA was isolated from purified embryos or mixed stages of wild-type strain N2 as described by Sambrook *et al*. [60], except that FastTrack columns (Invitrogen, San Diego, USA) were used (according to the manufacturer's protocols) instead of standard oligo(dT) columns. Likewise, a human fetal multiple tissue northern blot was probed with a portion of the *SCOCO *cDNA.

### Molecular biology

All manipulations were done following standard protocols [60]. A 1.5 kb *unc-69 *genomic fragment containing 700 bp upstream and 300 bp downstream of the coding region was engineered through site-directed mutagenesis to construct various amino- and carboxy-terminal CFP or GFP fusions (pgfpu69, pu69gfp and pSU083). The rescuing ability of the constructs was tested for both amino- and carboxy-terminal GFP fusions. A full-length *unc-69 *cDNA was cloned into pGEX-4T1 to generate a prokaryotic expression *unc-69-gst *construct. To generate a full-length *unc-76 *major splicing form cDNA (pSU001), the *Eco*RI-*Xba*I fragment of p76-c4 [14] was replaced by the *Eco*RI-*Bam*HI fragment of *yk784h09 *and the *Xba*I-*Bam*HI fragment of p76-c7, using three-way ligation. Progressive *unc-76 *deletions were made either by a PCR-based method, or using ExoIII and S1 nucleases following Erase-a-Base (Promega, Madison, USA) and ExoIII/S1 Deletion Kit (Fermentas, Hanover, USA) protocols. To generate genomic *unc-76 *constructs, an *Asc*I site was engineered 5' to the start ATG and was used to insert a 1 kb *Kpn*I-*Asc*I *unc-76 *promoter region up to the start codon. Subsequently a *Bgl*II-*Sph*I genomic fragment encompassing exons 1 to 3 was used to replace the corresponding region of the cDNA. An *Asc*I CFP cassette was inserted in-frame to create the amino-terminal CFP fusion plasmid (pSU065). Likewise, a *Not*I site was engineered immediately 5' to the stop codon to allow creation of the carboxy-terminal YFP fusion plasmid (pSU026). A genomic fragment containing the first 82 amino acids of mannosidase II was placed 3' to the *unc-69 *promoter and was carboxy-terminally fused with YFP to create pSU137. Full-length and truncated cDNAs of *unc-69*, *scoco*, *unc-76*, *arl-1*, *arl-3*, *arfrp*, and *unc-119 *were amplified from mixed-stage N2 mRNA pool or existing cDNA clones and subsequently subcloned into pRE192 and pRH143 to create LexA-bait and GAD-prey plasmids. pRE192 and pRH143 are derivatives of pBTM116 and pGAD424, respectively, and are gifts of K. Basler (University of Zurich).

Single-amino-acid changes were made following QuikChange Site-Directed Mutagenesis Kit protocols (Stratagene, La Jolla, USA). Sequences for each of the mutations are as follows: UNC-76(E275A): GAG→GCG; UNC-76(L281P): CTG→CCA; UNC-76(L287P): CTG→CCG; UNC-76(K291A): AAA→GCC; ARL-1(Q70L): CAA→CTA; ARFRP(Q79L): CAG→CTG. Mutations were confirmed by sequencing and swapped back into the original plasmids before further subcloning. All plasmids and construct sequences are available upon request.

### Protein sequence analysis

Prediction of coiled-coil structures was carried out using the COILS version 2.1 program [61]. The coiled-coil regions of UNC-69, UNC-76 and their homologs were assigned on the basis of a greater than 80% probability of forming coiled coils according to this program. We used ClustalW or T-Coffee for multiple sequence alignment and shaded the ClustalW alignment using BOXSHADE.

### Yeast two-hybrid

Random- and dT-primed yeast two-hybrid phage libraries were generous gifts from R. Barstead (Oklahoma Medical Research Foundation, Oklahoma City, USA). A further random-primed library was provided by M. Vidal (Harvard Medical School, Boston, USA). The *unc-69 *cDNA was subcloned into pDBTrp and pDBLeu vectors (PROQUEST Two-Hybrid System, GibcoBRL, Carlsbad, USA) and cotransformed with the AD plasmids. Transformed yeast reporter strain Mav203 (GibcoBRL) was patched onto plates lacking Leu and Trp (LW plates), and then replica plated onto plates lacking Leu, Trp and His with 75 mM 3-amino triazol (LWH/3AT plates), onto plates lacking uracil (URA plates) and onto filters for β-galactosidase assays. Only clones that activated all three (His, Ura, LacZ) reporter genes were kept for further analysis. Plasmid DNAs were purified from positive clones and retested for interaction with the bait plasmid. To study the interactions between UNC-69 and various ARL proteins, we used the yeast reporter strain L40, and followed Clontech's Yeast Protocols Handbook for *o*-nitrophenyl β-D-galactopyranoside (ONPG) and auxotrophic assays.

### *In vitro *translation and GST pull-down assay

*In vitro *transcription and translation of *unc-76 *was performed following protocols of the TNT-coupled reticulocyte lysate system (Promega) in the presence of ^35^S-labeled methionine (Amersham, Little Chalfont, UK). For pull-down experiments, purified recombinant GST and UNC-69-GST proteins were first immobilized on glutathione beads (Amersham). Immobilized proteins (2–10 μg) were incubated with 1–20 μl *in vitro *translated UNC-76 (depending on binding efficiency) in 1x interaction buffer (20 mM HEPES pH 7.9, 5 mM MgCl_2 _, 0.2% NP40, 0.2% BSA, 7.5% glycerol and protease inhibitor cocktail (Complete Mini, Roche, Indianapolis, USA)) at 4°C for 1.5–2 h. The beads were washed three times in washing buffer (100 mM KCl, 20 mM HEPES pH 7.9, 5 mM MgCl_2 _, 0.2% NP40 and protease inhibitor cocktail (Roche)), resuspended in 20 μl 2x SDS sample buffer, boiled and analyzed by SDS-PAGE and autoradiography.

### Germline transformations and array integration

For high-copy overexpression, plasmids were injected into the germline of adult hermaphrodites [62] at 50 ng/μl together with 150 ng/μl rescuing *dpy-20(+) *or pL15-EK *lin-15(+) *genomic fragments. To generate low-copy extrachromosomal arrays, plasmids were individually or co-injected each at 5 ng/μl together with 195 ng/μl pL15-EK. Transgenic progeny of injected animals were selected as non-Dpy or non-Muv animals at 25°C. Stably transmitting extrachromosomal arrays were integrated by γ-irradiation at 120 kV for 4.2 min.

### *In ovo *RNAi in chicken embryos

We obtained a cDNA clone of chick *SCOCO*, ChEST376p13, from the HGMP Research Center [63]. Double-stranded RNA for *in ovo *RNAi was transcribed *in vitro *as described [45]. Embryos were injected at stage 17–18 with 0.1 μl dsRNA (500 ng/μl) or a plasmid encoding YFP in PBS followed by electroporation. Stage 25–26 embryos [64] were stained and examined under a dissecting microscope with epifluorescence. With the site of injection chosen, we knocked down SCOCO only in MMC/LMC motor neurons, not in cells of the dermamyotome and/or the epaxial mesenchyme.

### Immunostaining of chicken embryos

For whole-mount immunostaining, staged embryos were dissected and fixed as described [45]. Embryos were permeabilized with 1% Triton X-100 in PBS, incubated in 20 mM lysine and 0.1 M sodium phosphate (pH 7.4), and in blocking solution (10% FCS in PBS) to reduce nonspecific staining. Mouse anti-neurofilament antibodies (RM0270, Zymed, San Francisco, USA) were used at 1:1,500 and applied for 48 h at 4°C. Embryos were washed in PBS at 4°C, and incubated in blocking solution before addition of secondary antibody (goat anti-mouse Cy3, 1:250; Jackson Laboratories, West Grove, USA). Embryos were dehydrated by sequential incubation in 25%, 50%, 75%, and 100% methanol. The tissue was cleared by transferring the embryos to BBBA (benzyl-benzoate:benzyl-alcohol = 2:1). The embryos were gently agitated until translucent, and were analyzed by fluorescent microscopy.

### *In situ *hybridization of chicken embryos

Embryos were sacrificed at different developmental stages (from stage 17 to 33) and fixed in 4% paraformaldehyde. The tissue was cryoprotected in 25% sucrose, and 20-μm-thick transverse sections of the lumbosacral spinal cord were used for the analysis of *SCOCO *expression as described previously [65].

## Additional data files

The following files are available: Supplementary results and tables (Additional data file 1); a figure showing that the overexpression of a full-length UNC-69(M1I)::GFP protein rescues locomotion defects of the *unc-69(e587) *mutants (Additional data file 2); a figure showing the extent of the *ok339 *deletion (Additional data file 3); and a figure showing that RNAi knockdown of chicken UNC-69/SCOCO results in epaxial nerve pathfinding defects (Additional data file 4).

## Supplementary Material

Additional data file 1 Supplementary results and tables Click here for additional data file

Additional data file 2 A figure showing that the overexpression of a full-length UNC-69(M1I)::GFP protein rescues locomotion defects of the *unc-69(e587) *mutants Click here for additional data file

Additional data file 3 A figure showing the extent of the *ok339 *deletion Click here for additional data file

Additional data file 4 A figure showing that RNAi knockdown of chicken UNC-69/SCOCO results in epaxial nerve pathfinding defects Click here for additional data file

## Figures and Tables

**Table 1:** Axon outgrowth and guidance defects in *unc-69 *mutants

	ALM defect (%)					
			
Genotype	B	NR	E	FL		*n *
Wild type	0	0	0	12		113
*unc-69(e602)*	12	36	77	84		77
*unc-69(e587)*	15	45	85	89		80
						
*unc-69(e602) *(m+z-)	0	4.3	39	91		70
*unc-69(e602)/nDf40 *(m+z-)	0	7.2	20	72		69
*unc-69(e587) *(m+z-)	1.4	7.2	43	87		69
*unc-69(e587)/nDf40 *(m+z-)	0	8.3	20	82		60
						
unc-69(e602)*	19	48	62	95		113
*unc-69(e602); opEx [P *_*mec *-7 _::unc-69]*	1	12	2	5		81
*unc-69(e602); opEx [P *_*mec *-7 _::unc-69]*	5	9	6	10		79
*unc-69(e602); opEx [P *_*mec *-7 _::unc-69]*	8	29	0	10		85
	AVM defect (%)					
		
Genotype	B	NR	E	FL	V	*n *

Wild type	0	0	1	8	0	106
*unc-69(e602) *	32	27	72	73	2.7	77
*unc-69(e587) *	64	70	86	87	0	80
						
*unc-69(e602) *(m+z-)	1.4	4.3	57	86	0	70
*unc-69(e602)/nDf40 *(m+z-)	0	0	56	80	0	69
*unc-69(e587) *(m+z-)	2.9	8.8	56	85	0	69
*unc-69(e587)/nDf40 *(m+z-)	0	3.6	76	95	0	60
						
unc-69(e602)*	46	67	93	100	ND	113
*unc-69(e602); opEx [P *_*mec *-7 _::unc-69]*	0	4	4	4	ND	81
*unc-69(e602); opEx [P *_*mec *-7 _::unc-69]*	8	12	12	23	ND	79
*unc-69(e602); opEx [P *_*mec *-7 _::unc-69]*	11	21	12	13	ND	85

Neurite outgrowth and guidance defects of mechanosensory touch neurons in *unc-69 *mutants. The morphology of neurites of ALM (top) and AVM (bottom) neurons (as in the schematic in Figure 2) was scored in different *unc-69 *mutants, in *unc-69/nDf40 *heterozygotes, and in mosaic animals carrying a functional *unc-69 *transgene under the control of the *mec-7 *promoter, which directs expression in the six touch neurons. All worms scored had a *P*_*mec*-4_*::gfp *transgene *zdIs5 *in the background to allow visualization of the neurite morphology. One ALM neurite was scored per animal. B, failure to form proper branch at the nerve ring; NR, failure of nerve ring branch to fully extend; E: failure to elongate past the branch point; FL, failure to extend fully; V, ventral guidance defect. (m+z-): homozygous mutant animals derived from heterozygous mothers. *These strains also carry a *lin-15(n765) *mutation in the background. All *opEx *transgenes also carry a wild-type copy of *lin-15(+) *as a coinjection marker. ND, not done. *n*, number of worms scored.

**Table 2:** Axon outgrowth and guidance defects of HSN, DD/VD, ALA and AVK neurons

Axon guidance phenotype	Defect in *unc-69(e587) *mutants (%)	*n *
HSN		
Ventral outgrowth	16	70
Midline crossover (HSNL)	38	40
Failure to reach nerve ring	99	59
DD/VD		
Dorsal outgrowth	33	45
ALA		
Premature termination	67	30
AVKR		
Premature termination or crossover	85	20

The morphology of HSN neurons was visualized using antibodies against serotonin; that of DD/VD neurons using antibodies against GABA; and that of ALA and AVKR neurons using antibodies against FMRF-amide. See Materials and methods for details. *n*, number of animals scored.

**Table 3:** Quantitative analysis of axon extension defects in *unc-69(lf)*, *unc-76(lf) *and other mutants

Genotype	2 AWC^OFF ^(%)	1 AWC^ON ^(%)	2 AWC^ON ^(%)	*n *
Wild type	1	99	0	442
				
*unc-69 *				
*unc-69(ju69)*	1	99	0	190
*unc-69(e602)*	34	66	0	119
*unc-69(e587)*	30	70	0	194
				
*unc-76 *				
*unc-76(rh116)*	11	89	0	83
*unc-76(n2457)*	6	94	0	102
*unc-76(n2397)*	8	92	0	64
*unc-76(ev424)*	10	90	0	68
*unc-76(n2367)*	30	70	0	84
*unc-76(n2399)*	25	75	0	67
*unc-76(e911)*	47	53	0	101
*unc-76(e911); lon-2(e678)*	31	69	0	91
*unc-76(n2398)*	28	72	0	184
				
Double mutants				
*unc-69(e602); unc-76(n2457)*	35	65	0	106
*unc-69(e602); unc-76(e911)*	48	52	0	118
*unc-69(e587); unc-76(n2457)*	33	67	0	108
*unc-69(e587); unc-76(e911)*	31	69	0	143
				
Other axonal guidance mutants				
*sax-3(ky123)*	64	33	3	112
*lon-2(e678) unc-6(n102)*	38	62	0	65
*vab-3(e648)*	54	40	6	68
*unc-33(e204)*	7	73	20	135
*unc-119(ed3)*	12	48	39	99
				
Other double mutants				
*unc-76(e911); sax-3(ky123)*	95	5	0	22
*unc-76(e911); lon-2(e678) unc-6(n102)*	73	27	0	152
*unc-76(e911); vab-3(e648)*	63	27	10	62
*unc-33(e204); unc-76(e911)*	80	18	1	291
*unc-119(ed3); unc-76(e911)*	65	32	3	167

All animals scored had *kyIs140 *(*P*_*str*-2_*::gfp) *in the background, which turns on its expression in only one of the two AWC neurons (1 AWC^ON^) in wild-type animals. In axon guidance mutants, *P*_*str*-2_*::gfp *expression is silenced in both AWCs (2 AWC^OFF^) owing to failure of axonal contact. All *unc-69 *and *unc-76 *alleles except *unc-76(rh116) *are arranged in order according to their physical position (5' to 3') in the open reading frame. *n*, number of animals scored.
