# Supplementary material for: The short coiled-coil domain-containing protein UNC-69 cooperates with UNC-76 to regulate axonal outgrowth and normal presynaptic organization in Caenorhabditis elegans
Source: J Biol. 2006 May 25;5(4):9. doi: 10.1186/jbiol39 (PMC1561584; doi:10.1186/jbiol39)
Supplement: Additional data file 1 — Supplementary results and tables [file jbiol39-s1.pdf]

Additional data file 1

Supplemental Results

unc-69(ok339) deletion

unc-69(ok339) deletes a 2.65 kb genomic fragment encompassing the whole *unc-69* transcription unit as well as flanking sequences both 5′ and 3′ of the gene (Supplemental Figure S1). Thus, this deletion is certain to represent a null allele of *unc-69*. *unc-69(ok339)* homozygotes have an Unc phenotype and arrest during L1 to L2 transition (Supp. Table S2). We found that *ok339* also deletes *T07A5.5* (predicted to encode the *C. elegans* homolog of the Ost4p subunit of the *S. cerevisiae* oligosaccharyltransferase) and ends but 200 bp 5′ of the *unc-50* coding region (unpublished). We reasoned that the *ok339* arrest phenotype could either be due to loss of *T07A5.5*, or a synthetic phenotype due to the simultaneous loss of both *unc-50* and *unc-69* function. Indeed, *unc-69(ok339)* mutant worms were resistant to 25mM levamisole, a hallmark of *unc-50* mutations. Furthermore the *ok339* deletion failed to complement *unc-50(e306)* mutants (data not shown). We balanced *unc-69(ok339)* with *qC1*, and microinjected a 6.7 kb Hind III-EcoR I genomic fragment (*pUnc50-10*) carrying wild-type copies of both *unc-50* and *T07A5.5* into the deletion carrying strain. Transgenic worms homozygous for *unc-69(ok339)* grew to adulthood but were sterile (three independent lines). It is likely that the sterility is due to lack of germline expression of *T07A5.5* off the extrachromosomal array. For this reason, we did not pursue usage of *unc-69(ok339)* in our studies.

The *unc-69* locus encodes multiple splice variants

We found a splice variant of *unc-69* by sequencing EST clones provided by Yuji Kohara (National Institute of Genetics, Japan), which we termed *T07A5.6b* (GenBank accession number AY919833). This splice variant (encoded by *yk508g10*) adds another 14 amino acids (DDSVHD-DDFGEYEY) to the carboxyl terminus of UNC-69. The small peptide is enriched in acidic amino acid residues, and makes the carboxyl terminus quite acidic (Figure 1a,b). Wormbase also predicts the existence of splice variant *T07C4.10a*, which could encode a 1138-amino-acid protein. We have failed to obtain any experimental evidence to support the existence of this latter splice variant. It is likely that *T07C4.10a* is either expressed at a very low level or is improperly predicted. Therefore we removed the *unc-69* coding sequence from *T07C4.10a* and renamed the remaining coding sequence as *T07C4.10* (Supp. Figure S1; also see WormBase [55]).

UNC-69 homologs

A *C. elegans unc-69* cDNA was used to probe a *C. briggsae* genomic library (gift of D. Baillie) in lambda Charon4 under low stringency conditions (hybridization at 55°C in 6x SSPE, 0.5% SDS, washing at 55°C, twice in 2x SSPE, 0.5% SDS, and twice in 0.5x SSPE, 0.5% SDS). Positive phage were purified and EcoR I insert fragments were subcloned into pBluescript, and their DNA sequence determined. We also identified hybridizing bands in *C. vulgaris, C. remanei,* and *Ascaris suum.* Additional UNC-69-like proteins were found in the expressed sequence tag (EST) database of rat (CB577413) and chimpanzee (AU298017). Partial UNC-69 sequences were also found in chick EST collections (BU311615). Surprisingly, the primary amino-acid sequences or rat, mouse, chimpanzee and human are 100% identical.

Homophilic interaction of UNC-69

In many proteins, a coiled coil domain mediates homophilic dimer- or trimer- formation. We thus used the Y2H system to assay UNC-69's ability to interact with itself. We created yeast GAL4 DNA binding domain (DB)- and activation domain (AD)-UNC-69 fusion constructs, and transformed them into the yeast reporter strain HF7c. A very weak expression of the His reporter, as assayed by growth on LWH-plates, was observed just above background level. However the interaction was not strong enough to activate the LacZ reporter above background level (data not shown).

Supplemental Table S1. *unc-69* mutants lay more eggs in the absence of food than wild-type animals

	Eggs laid (M9 assay)			Eggs laid (plate assay)		
Genotype	M9	M9 + 5 mg/ml 5-HT	*n*	- Food	+ Food	*n*
Wild type	0	26	5	0	30	5
unc-69(e587)	13	ND	5	16	28	5
unc-69(e602)	14	ND	5	5	11	5

Total number of eggs laid in 60 min. (M9 assay) or 90 min. (plate assay) was determined. *n*, number of animals tested. ND, not done.

Supplemental Table S2. *unc-69(ok339)* mutants arrest development as L1 larvae

Genotype	Eggs laid per animal	Progeny		*n*
		Hatching (%)	L1 arrest (%)*	
qC1/unc-69(ok339)	157±26	99.9±0.9	24.2±4.1	9

*L1 arrest (%) represents per cent of hatched progeny that failed to develop past the first larval (L1) stage within six days of hatching (wild-type larvae remain in the L1 stage for about 12 h at 20°C). L1 arrested larvae were confirmed to be *unc-69(ok339)* homozygotes by nested PCR. Data are mean±s.d. *n*, number of broods analyzed.

We also assayed for a homophilic interaction of UNC-69 *in vitro* in several different ways. Maltose-binding protein (MBP)-tagged and GST-tagged UNC-69 were expressed in bacteria and used in pull-down assays using column of beads against either one or the other of the fusion proteins followed by western analysis. We were not able to observe any interaction between GST-UNC-69 and MBP-UNC-69 (data not shown). We also *in vitro* translated S-tagged and T7-tagged UNC-69 proteins separately, mixed the proteins at room temperature, immunoprecipitated with anti-T7 beads followed by western blots using antibodies against the S-tag. No interaction between T7-UNC-69 and S-UNC-69 was observed, even when the conditions were extremely mild. Finally we failed to observe any homophilic interaction of UNC-69 when T7-UNC-69 and S-UNC-69 were cotranslated *in vitro* (data not shown). These results suggest that UNC-69 likely does not interact with itself.

unc-76 interacts genetically with *unc-16* and *vab-8*

In *C. elegans*, UNC-16 associates with the RUN-domain protein UNC-14 and the Kinesin–1 light chain KLC-2 [9]. In addition, UNC-14, a serine/threonine kinase UNC-51, and VAB-8 associate with each other to regulate axonal outgrowth [10]. To explore possible genetic interactions between these Kinesin-1-interacting proteins and the UNC-69-UNC-76 complex, we assayed AWC axon extension defects in different double mutant backgrounds.

We first analyzed AWC axon extension defects in *unc-116(e2310); unc-76(e911)* double mutants. The *unc-116(e2310)* mutation only caused very mild axon extension defect on its own. However, like other axon guidance mutants, *unc-116(e2310)* significantly enhanced the AWC axon extension defects of *unc-76(e911)* mutants (Supp. Table S3).

We then assayed AWC axon extension defects in various *unc-16; unc-76, unc-76 unc-51*, and *vab-8 unc-76* double mutant backgrounds (for technical reasons we did not analyze *unc-14; unc-76* double mutants). The *unc-51(e369)* mutation resulted in a very mild defect, and did not enhance AWC axon extension defects caused by the *unc-76(e911)* mutation (Supp. Table S3). Surprisingly, mutations in *unc-16* and *vab-8*, when introduced into a *unc-76(e911)* background, either slightly or significantly suppressed the AWC axon extension defects (Supp. Table S3). Thus, *unc-76* shows intriguing patterns of genetic interactions with both *unc-16* and *vab-8* in AWC neuronal fate determination. However, our data do not indicate if UNC-16 and VAB-8 directly participate in the UNC-76-UNC-69-dependent AWC axon extension processes. The relationship between UNC-76, UNC-16 and VAB-8 will thus need to be further analyzed.

Supplemental Table S3. *unc-16* and *vab-8* mutations suppress the AWC axon extension defect of *unc-76(e911)* mutant.

Genotype	2 AWC^{OFF}(%)	1 AWC^{ON}(%)	2 AWC^{ON}(%)	*n*
Wild type	1	99	0	442
unc-76(e911)	47	53	0	101
unc-116(e2310)	2	98	0	111
unc-16(ju146)	0	100	0	76
unc-16(e109)	1	99	0	104
unc-51(e369) rol-9(sc148)	1	98	1	90
vab-8(ev411)	0	100	0	61
vab-8(gm84)	1	99	0	70
unc-116(e2310); unc-76(e911)	66	34	0	151
unc-16(ju146); unc-76(e911)	12	88	0	197
unc-16(e109); unc-76(e911)	4	84	12	197
unc-76(e911) unc-51(e369) rol-9(sc148)	46	54	0	219
vab-8(ev411) unc-76(e911)	30	70	0	130
vab-8(gm84) unc-76(e911)	28	72	0	301

All animals scored had *kyIs140* (*P_{str-2}::gfp*) in the background. *n:* number of animals scored.
