# Supplementary material for: The short coiled-coil domain-containing protein UNC-69 cooperates with UNC-76 to regulate axonal outgrowth and normal presynaptic organization in Caenorhabditis elegans
Source: J Biol. 2006 May 25;5(4):9. doi: 10.1186/jbiol39 (PMC1561584; doi:10.1186/jbiol39)
Supplement: Additional data file 2 — A figure showing that the overexpression of a full-length UNC-69(M1I)::GFP protein rescues locomotion defects of the unc-69(e587) mutants [file jbiol39-s2.pdf]

Additional data file 2

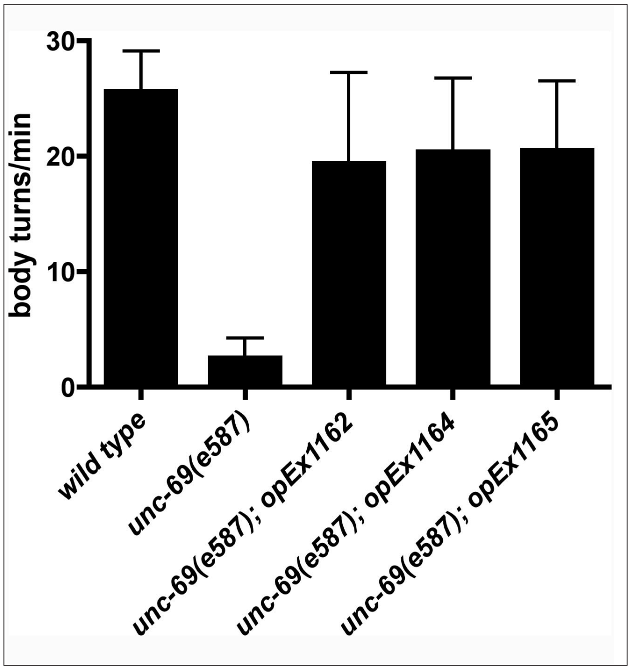

Supplemental Figure S1 (Additional data file 2)
Overexpression of a full-length UNC-69(M11)::GFP protein rescues locomotion defects of the *unc-69(e587)* mutants. pSU86 (*Punc-69::unc-69(M11)::gfp)* was microinjected at 50 ng/ml into *unc-69(e587); lin-15(n765ts)* mutant hermaphrodites. Non-Muv transgenic hermaphrodites were selected for further characterization. Three independent transgenic lines were assayed for locomotion improvement. For wild type, *n* = 14. For the other genotypes, *n* = 10. Data shown represent mean ± S.D. Note that all transgenic lines also carry the *lin-15(n765ts)* mutation in the background.
