# Supplementary material for: The short coiled-coil domain-containing protein UNC-69 cooperates with UNC-76 to regulate axonal outgrowth and normal presynaptic organization in Caenorhabditis elegans
Source: J Biol. 2006 May 25;5(4):9. doi: 10.1186/jbiol39 (PMC1561584; doi:10.1186/jbiol39)
Supplement: Additional data file 3 — A figure showing the extent of the ok339 deletion [file jbiol39-s3.pdf]

Additional data file 3

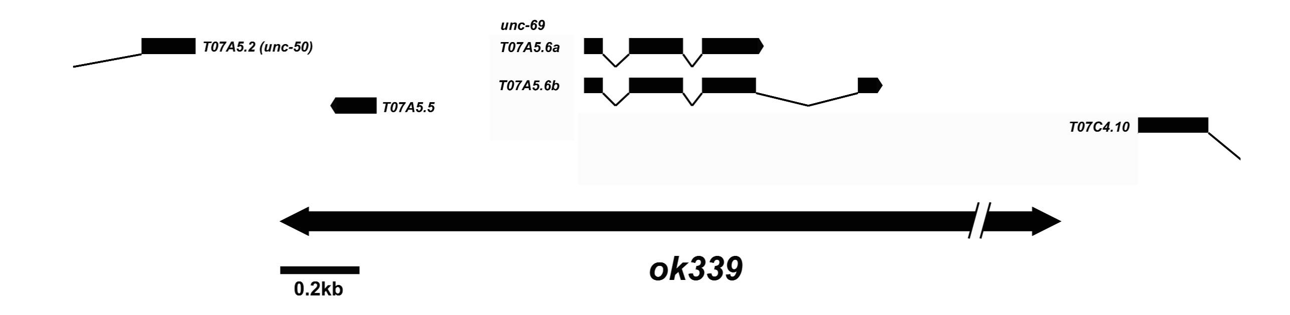

Supplemental Figure S2 (Additional data file 3)
Extent of the *ok339* deletion. *ok339* deletes a 2.65 kb genomic region containing both verified splice variants of *unc-69*, as well as the predicted single-exon gene *T07A5.5*. Deletion breakpoints: 5′-TAAATTAGGG/tagagacgaa … 2.65 kb … gtcacggtgt/TCACACGTTT-3′.
