# Supplementary material for: The short coiled-coil domain-containing protein UNC-69 cooperates with UNC-76 to regulate axonal outgrowth and normal presynaptic organization in Caenorhabditis elegans
Source: J Biol. 2006 May 25;5(4):9. doi: 10.1186/jbiol39 (PMC1561584; doi:10.1186/jbiol39)
Supplement: Additional data file 4 — A figure showing that RNAi knockdown of chicken UNC-69/SCOCO results in epaxial nerve pathfinding defects [file jbiol39-s4.pdf]

Additional data file 4

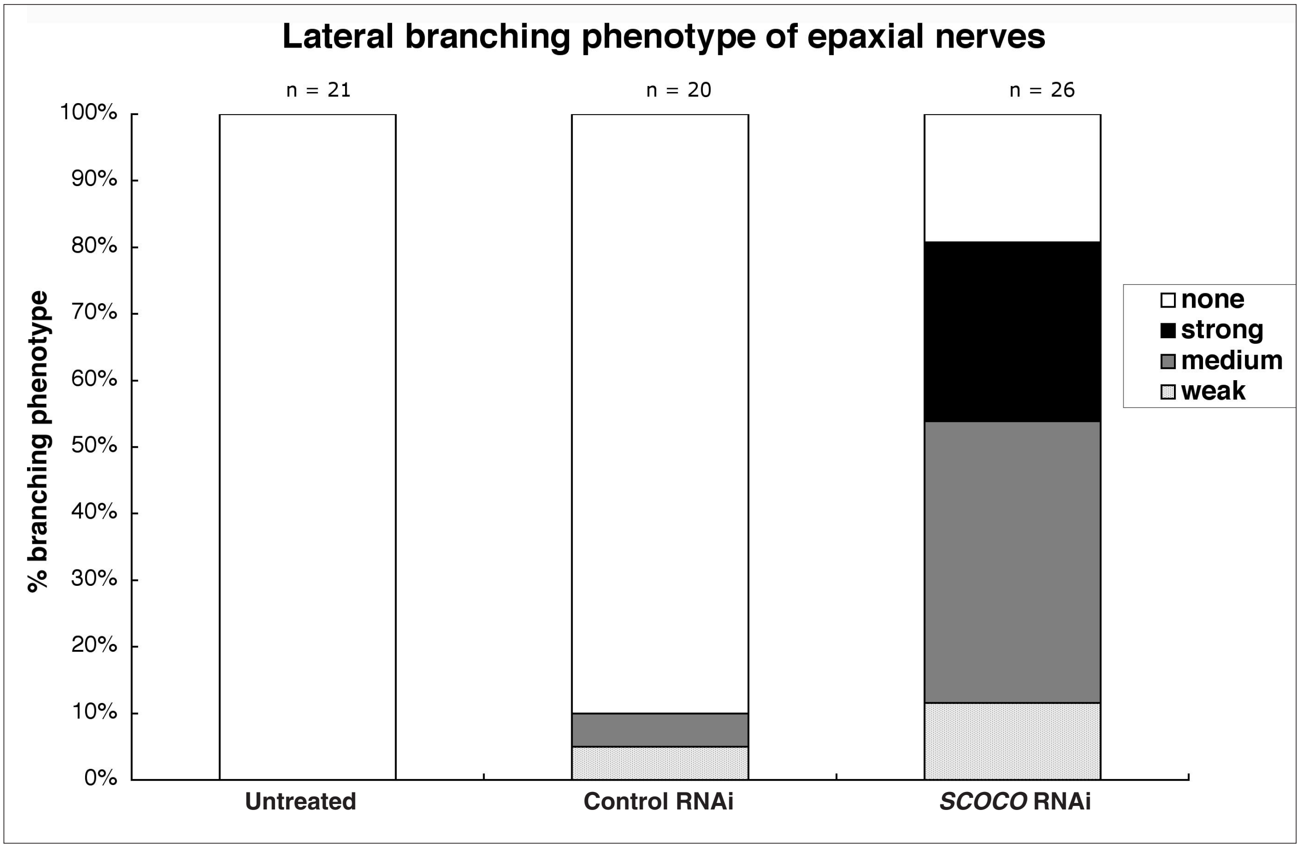

Supplemental Figure S3 (Additional data file 4)
RNAi knockdown of chicken UNC-69/SCOCO results in epaxial nerve pathfinding defects. Untreated, control (*yfp*-RNAi)-treated, and *SCOCO* RNAi-treated chicken embryos were scored for epaxial nerve defects. Embryos were classified into four groups, depending on whether they showed a strong, medium, weak, or no epaxial nerve defect.
